# Markerless On-Device Detection of Compensatory Movement Patterns in Upper-Limb Rehabilitation Exercises from Monocular RGB Video: A Validation Study in Healthy Adults

**DOI:** 10.3390/s26165054

**Published:** 2026-08-09

**Authors:** Artem Pavlikov, Vera Petrosyan, Vladislav Agapov, Mikhail Gorodnichev, Danila Lobunko, Dmitry Skvortsov

**Affiliations:** 1 Faculty of Information Technologies, Moscow Technical University of Communications and Informatics (MTUCI), Aviamotornaya St. 8A, Moscow 111024, Russia; v.r.petrosyan@mtuci.ru (V.P.); v.a.agapov@mtuci.ru (V.A.); m.g.gorodnichev@mtuci.ru (M.G.); 2 Federal Center of Brain Research and Neurotechnologies of the Federal Medical Biological Agency (FMBA of Russia), Ostrovityanova St. 1, Bldg. 10, Moscow 117513, Russiaskvortsov.biom@gmail.com (D.S.)

**Keywords:** human pose estimation, action recognition, rehabilitation, compensatory movement, markerless motion analysis, on-device inference, edge computing, telerehabilitation, computer vision, IMU validation

## Abstract

Neurological disorders drive demand for prolonged upper-limb rehabilitation, yet specialist access is uneven and assessment stays subjective. Marker-based and inertial measurement unit (IMU) systems are accurate but costly and impractical at home, while pose estimation pipelines mostly stop at keypoints, and many process video server-side, raising privacy concerns. We present a markerless pipeline that analyzes monocular RGB video entirely on-device in the browser, so it never leaves the machine. From 33 BlazePose keypoints, it derives five geometric metrics designed to limit body-size dependence—incomplete elbow extension, inter-limb asymmetry, shoulder girdle elevation, lateral trunk lean, and head tilt—compared against empirically calibrated, preliminary thresholds; a finite-state machine segments repetitions, and the flags are pooled into an unvalidated, exploratory quality score. Against an IMU reference over the 0–$62^{\circ }$ range that the recordings cover, the image-plane angle showed a mean absolute error of $2.18^{\circ }$, below the pre-specified $5^{\circ }$ tolerance, a trajectory-averaged bias within $\pm 2^{\circ }$, and Lin’s concordance correlation coefficient of 0.956; the difference is, however, proportional to the angle—about 4% of the measured value—so the accuracy should not be extrapolated to larger elevations, and because that comparison was made offline, it does not include the timing error of the causal real-time path. On a single seated frontal-plane abduction task, with 18 healthy volunteers simulating the compensations and annotated by two independent clinicians blind to the instructed condition, compensation detection reached a macro-averaged $F_{1}$ of 0.75 and 0.72 against the individual raters. The five signs differ in maturity: near-expert for trunk lean and head tilt, moderate for incomplete elbow extension and inter-limb asymmetry, and weakest for shoulder elevation, which a single frontal view cannot fully disentangle from the abduction motion. Running at 22–30 frames per second on consumer laptops without relying on a discrete GPU, it offers an accessible, privacy-preserving proof-of-concept foundation for home telerehabilitation; generalization beyond this one exercise and effectiveness on genuine post-stroke compensations remain to be established.

## 1. Introduction

Neurological disorders constitute a growing global health burden: the Global Burden of Disease analysis reports a sustained rise in disability-adjusted life years attributable to conditions of the nervous system, including stroke, neonatal encephalopathy, and Alzheimer’s disease, driven by population growth and aging and by the increasing prevalence of disabling sequelae [1]. Survivors of such conditions require prolonged, adaptive rehabilitation that depends on individualized programs and the continuous involvement of trained specialists [2,3]. Yet rehabilitation resources remain unevenly distributed: specialized centers are concentrated in major urban areas, so that populations in remote regions face a shortage of qualified therapists and patients with limited mobility face considerable difficulty in reaching them. This access gap motivates telerehabilitation, in which automated tools extend therapy beyond the clinic [4]. Such tools can reduce the workload on clinical staff, sustain patient motivation, and provide objective, continuous monitoring of recovery provided that they are accurate, accessible, and easy to use at home [5]. What such programs still lack is a measure of how the exercise is actually performed. Because home practice is unsupervised, exercise fidelity—the extent to which the patient performs the movement the provider intended, as opposed to merely completing a session—is difficult to assess, correct, or monitor; a recent scoping review surveyed the measures used for it in home-based cardiovascular rehabilitation [6].

Clinical assessment of motor function, however, remains difficult to perform objectively and continuously. Standardized clinical scales such as the Fugl–Meyer Assessment, the Barthel Index, and the Ashworth scale are widely used, but their resolution is bounded by subjective classification: the score noise is comparable to a single scale step, so changes smaller than one point are difficult to distinguish, and ceiling effects further limit sensitivity [7]. Manual goniometry and visual inspection are similarly subjective and cannot track movement dynamics in real time with typical clinical errors of 3–$4^{\circ }$ that vary between raters [8,9]. The difficulty is sharper for compensatory movement, which is judged qualitatively rather than measured: when 22 experienced therapists rated video recordings of a standardized drinking task, inter-rater agreement ranged from good for individual task phases (ICC=0.88) down to poor for global movement quality (ICC=0.40), and the authors did not recommend such ratings as a ground truth without dedicated rater training and calibration [10]. Instrumented references are far more reliable—electro-goniometers achieve high test–retest reliability (ICC≈0.9) [9], and marker-based optical stereophotogrammetry achieves sub-millimeter spatial accuracy at 60–240 Hz [11,12]—but they demand controlled laboratory environments, are sensitive to occlusion and to soft-tissue artifacts of the shoulder girdle [13], and their cost and low portability make them impractical for home telerehabilitation. Wearable inertial measurement units (IMUs) remove the line-of-sight constraint, yet a full-body model requires many synchronized sensors and remains expensive and burdensome to calibrate, which limits routine unsupervised use at home.

By contrast, markerless human pose estimation (HPE) from ordinary RGB cameras offers an accurate yet accessible way to recover biomechanical movement parameters, making it attractive for both home and clinical settings [14,15]. Recent validation studies show that deep HPE networks can approach the optoelectronic standard: MediaPipe-based joint-angle measurements agree with goniometric reference to within about $2.5^{\circ }$ [16], while a low-cost depth camera reaches about $4.1^{\circ }$ against a marker-based system during dynamic movement [17]. The evidence has broadened considerably in the last few years: BlazePose-based gait analysis now agrees closely with multi-camera optical capture [18], MediaPipe has been used to track upper-limb reaching movements in stroke survivors [19], stereo and depth fusion approaches report marker-comparable accuracy for selected exercise parameters [20,21], and dedicated benchmarks now evaluate pose methods specifically on rehabilitation exercises [22], on hand and finger movement [23], and on the limb range of motion from a single camera [24]. The most recent evidence is at once more focused on the upper limb and more qualified about where markerless capture holds: shoulder and elbow kinematics are reproduced against an optoelectronic reference with intraclass correlations above 0.91, yet maximum flexion is systematically underestimated where occlusion and joint geometry interfere [25]; agreement between a webcam BlazePose pipeline and an optoelectronic reference varies sharply with the motion examined, remaining poor for shoulder flexion (ICC=0.18) and only moderate for elbow flexion (ICC=0.53), which the authors attribute to reduced landmark sensitivity near the end of range [26]; a monocular assessment of reachable workspace agrees closely with a marker-based reference from a frontal view but degrades as the camera moves off axis [27]; in patients with shoulder and elbow pathology, single-camera estimation matches or exceeds expert visual assessment for elbow flexion while falling short of it for extension [28]; and a benchmark of sixteen monocular frameworks on exercise video finds that joint-angle accuracy still differs substantially between methods [29]. Available architectures span the accuracy–efficiency trade-off: OpenPose remains a high-accuracy offline reference but needs a discrete accelerator for real-time inference [30]; the lightweight, browser-capable PoseNet [31] and the single-stage MoveNet [32] run on mobile devices but use a 17-keypoint topology without hand or foot detail; and MediaPipe Pose (BlazePose) directly regresses 33 three-dimensional keypoints from a compact MobileNet backbone at 20–30 ms per frame on mobile devices with per-landmark visibility scores [33,34]. The upper-limb references our metrics need are present in all three lightweight backbones; the choice among them turns on tracking stability and the in-browser runtime rather than on landmark count alone. Despite this progress, most pipelines stop at keypoint extraction rather than at an interpretable detection of clinically relevant compensatory patterns such as trunk lean, shoulder elevation, and inter-limb asymmetry [35,36,37], and many run server-side, which raises privacy and connectivity concerns for medical video. A fully client-side pipeline instead keeps identifiable video on the device—exporting only derived data (keypoints and metrics), never the raw image stream—in line with the broader shift toward privacy-preserving processing of medical data [38]. To the best of our knowledge, existing work achieves only subsets of these properties: a browser-based upper-limb telerehabilitation system that unites on-device in-browser execution, reproducible compensation metrics with fixed thresholds (designed to limit anthropometric dependence) and an aggregate quality score, and validation in two parts—the arm-elevation angle against an IMU equivalence reference and the compensation detector against blinded clinician ratings—remains absent.

We are explicit about the nature of the contribution. The individual building blocks—the BlazePose backbone, geometric joint-angle descriptors, finite-state repetition segmentation, and fixed-threshold rules—are established techniques, and we do not claim a new pose-estimation or machine-learning method. The novelty is instead one of *system integration and validation*: assembling these components into a fully on-device, browser-based compensation-detection loop that meets a real-time budget on commodity hardware (an engineering contribution) and quantifying what that assembled system can and cannot measure through an IMU-referenced angle study and a blinded two-clinician annotation study (an empirical contribution). With that framing, there are four main contributions:

- A markerless, fully on-device (in-browser) pipeline that estimates upper-limb kinematics from a single RGB camera and runs the entire “frame → pose → metrics → feedback” loop locally so that the raw video never leaves the device and only derived data (keypoints and metrics) may be exported.
- A minimal, reproducible set of five normalized geometric metrics, designed to limit body-size dependence, that quantify the most common compensatory patterns of the shoulder girdle with fixed, empirically calibrated thresholds and an aggregate quality score *Q* that summarizes overall execution quality (its constituent per-repetition flags, not *Q* itself, are validated).
- Two-part validation with deliberately separated scope: the arm-elevation angle against a synchronized IMU reference (MAE $2.18^{\circ }$, Lin’s concordance correlation coefficient 0.956), and the five compensation flags—the only evidence for the four non-angular metrics—against blinded annotation by two clinicians (macro-averaged $F_{1}$ of 0.75 and 0.72 per rater). The signs differ in maturity, so the macro-average is not a single deployable-performance figure.
- A characterization of real-time feasibility on commodity hardware at 22–30 FPS across six consumer laptops without relying on a discrete GPU.

The remainder of this paper is organized as follows. Section 2 reviews related work on markerless pose estimation and compensation analysis; Section 3 details the proposed metrics and the client-side pipeline; and Section 4 reports the validation experiments and real-time performance. Section 5 discusses the findings and limitations, and Section 6 concludes.

## 2. Related Work

### 2.1. Human Pose Estimation Models and Clinical Suitability

Markerless HPE architectures span a broad accuracy–efficiency trade-off that directly governs their suitability for unsupervised home use. OpenPose pioneered bottom -up multi-person estimation by coupling confidence heatmaps with part affinity fields, and it remains a high-accuracy reference for offline biomechanical analysis; sustained real-time inference, however, depends on a discrete GPU, which makes it impractical as the core of an interactive, CPU-only telerehabilitation service [30]. Lighter single-person models were designed for exactly this constraint: PoseNet pairs a MobileNet backbone with heatmap-plus-offset decoding to run inside a browser, but in its single-person configuration, it uses a seventeen-keypoint topology that omits the interphalangeal and distal landmarks needed for fine-motor assessment [31,34], while MoveNet replaces iterative refinement with a single-stage regression head that minimizes branching and latency at a comparable topology [32]. BlazePose (MediaPipe Pose) instead regresses thirty-three three-dimensional landmarks—including facial, hand, and foot points—from a compact MobileNet-derived backbone, tracking landmarks across frames rather than re-detecting each frame independently, which stabilizes the skeleton under partial occlusion [33]. It should be noted that the standard seventeen-keypoint (COCO) topology of PoseNet and MoveNet already includes the ears, shoulders, elbows, wrists, and hips, which are exactly the landmarks our five metrics require; the choice of BlazePose is therefore not forced by missing anatomy but by implementation-level properties that matter for an interactive, CPU-only browser deployment. In practice, we prefer it for its frame-to-frame tracking stability and the maturity of its in-browser runtime rather than for topology alone. A more recent line of work replaces convolutional backbones with vision transformers—TransPose uses attention to expose the spatial dependencies behind keypoint localization [39], and ViTPose shows that plain transformer encoders set a strong accuracy baseline for pose estimation [40]. These models raise the accuracy ceiling but carry a heavier compute and memory footprint than is currently practical for real-time, CPU-only inference inside a browser, which is why we retain a lightweight CNN backbone; distilling a transformer estimator to that budget is an appealing future direction. Single-camera markerless capture has also been assessed against a marker-based reference during upper-limb activities of daily living by driving an inverse-kinematic musculoskeletal model with the estimated keypoints; agreement there depends strongly on the angle measured with a root-mean-square error near $8^{\circ }$ for shoulder elevation but around $21^{\circ }$ for shoulder rotation [41]—one reason this paper reports angles in the movement plane rather than axial rotations. A residual limitation is clinically important: BlazePose and its peers are trained predominantly on able-bodied subjects, so atypical post-stroke trajectories or limb deformities can cause the estimator to lock onto a neighboring heatmap maximum and produce coordinate jumps, which motivates normalized descriptors computed on top of the raw landmarks rather than reliance on the coordinates alone.

### 2.2. Computer Vision for Rehabilitation and Compensation Detection

A growing body of validation studies confirms that markerless HPE can reach measurement-grade accuracy for rehabilitation kinematics; a systematic review of markerless motion capture for clinical measurement in rehabilitation maps both the breadth of this evidence and the areas where it remains thin [42]. BlazePose-based gait analysis agrees closely with a multi-camera Vicon system [18], MediaPipe has been applied to upper-limb reaching in stroke survivors [19], and multi-view pipelines that fuse pose with depth—whether YOLOv8x-pose with a stereo depth camera against OptiTrack [20] or stereo information fusion over MediaPipe Pose, which lowers reconstruction error from 56.3 to 30.1 mm relative to monocular input [21]—approach marker-based accuracy for selected gait and exercise parameters. Dedicated benchmarks and datasets now compare HPE methods specifically on physical-rehabilitation exercises [22], on hand and finger movement against marker-based capture [23], and on limb range of motion from a single camera versus goniometry [24]. These studies share a common boundary: they quantify joint angles, range of motion, and symmetry with high fidelity, yet they stop at kinematic measurement and do not formalize the reproducible detection of compensatory patterns.

Compensation detection itself has been pursued mainly through instrumented, non-RGB modalities. Pressure-distribution mats with handcrafted features and classical classifiers discriminate trunk lean, trunk rotation, and scapular elevation in stroke survivors [43], and the same signal driven online inside a rehabilitation robot reduces measured compensation in a closed loop [44]; wearable inertial sensors with machine learning provide quantitative compensation scores in post-stroke patients [45]. Depth- and skeleton-based vision has likewise been used: a Kinect virtual-rehabilitation system applies fixed thresholds with audio-visual feedback to flag compensation [46], and sequence models—RNN, GRU, LSTM, and Transformer—classify compensation on the Kinect-recorded Toronto Rehab Stroke Pose dataset [47]. Closest to our modality, recent computer-vision work assesses asymmetry and compensatory deviations in physiotherapy directly from RGB video using a pose detector [48]. Also in this modality, a feasibility study detected shoulder compensation in stroke survivors from consumer-grade webcams using deep networks over pose keypoints, and it reported the gap that matters most for deployment: an accuracy of 0.92–0.95 when training and testing within the same participants fell to about 0.70 when generalizing to unseen ones [49]. These approaches, however, mostly depend on special hardware (pressure seats, robots, IMUs, or depth cameras) or on data-driven classifiers whose thresholds are learned per setup and may not transfer across subjects or acquisition conditions without re-tuning. The threshold-based work in our own modality is closest in spirit but does not close the gap: its compensation scoring rests on hand-set angle bins that are neither fitted to data nor evaluated, and the work reports keypoint-localization accuracy without any compensation-detection result [48]. None of these works, then, offers a published set of compensation metrics with fixed thresholds designed to limit anthropometric dependence together with a reported detection performance against an independent observer.

### 2.3. On-Device Inference and Privacy in Medical Video

Where these pipelines execute matters for telerehabilitation as much as their accuracy. Lightweight architectures already make pose inference feasible on edge hardware: limb range-of-motion analysis has been demonstrated on resource-constrained edge devices from a single monocular camera [24], and MobileNet-class backbones keep BlazePose within a real-time budget on commodity phones [33,34]. Running the estimator server-side, by contrast, requires transmitting identifiable patient video off the device, which introduces latency, a hard connectivity dependency, and privacy exposure that are particularly problematic for medical data; keeping computation local so that the raw image stream never leaves the device—only derived data may be exported—aligns with the broader move toward the privacy-preserving processing of health information [38]. A fully client-side, in-browser loop that performs frame capture, pose estimation, and metric computation on the user’s own device therefore removes both the network dependency and the need to store raw video remotely.

Table 1 positions the present system against the closest prior compensation-analysis approaches on the axes that matter for unsupervised home use.

Prior work delivers either accurate markerless kinematics without reproducible compensation detection or compensation detection that is tied to specialized hardware and per-setup learned thresholds, while on-device privacy is rarely treated as a first-class design goal. To our knowledge, no prior system has united markerless monocular-RGB capture, fully in-browser on-device execution, and a compact set of compensation metrics with empirically calibrated preliminary thresholds (designed to limit anthropometric dependence) and an aggregate quality score with the arm-elevation angle validated against an IMU equivalence reference and the compensation detector benchmarked against blinded clinician ratings. This paper targets precisely this gap; its contribution is the integration of these elements into a working on-device system and their empirical validation rather than a new pose-estimation method. As Section 5 sets out, that validation establishes measurement adequacy on healthy volunteers, not clinical effectiveness, and the five signs differ in maturity.

## 3. Materials and Methods

### 3.1. Pipeline Overview

**Figure 1:**
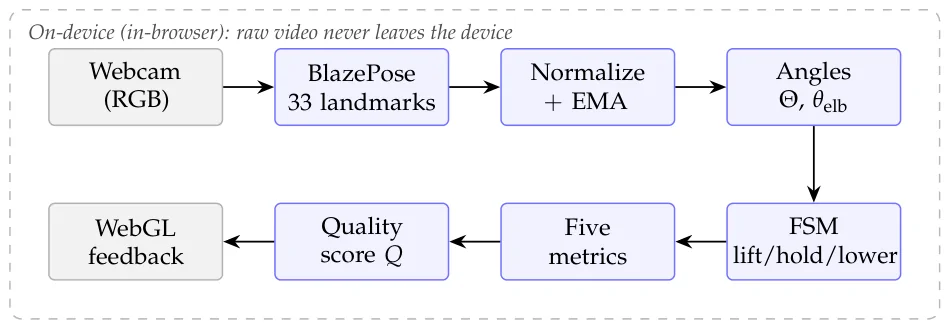
Overview of the proposed fully on-device markerless pipeline. A frontal monocular RGB stream is captured in the browser via WebRTC and processed by BlazePose (33 landmarks); after coordinate normalization and exponential smoothing, the arm-elevation angle Θ(t) and elbow angle $\theta_{\mathrm{elb}}\left(t\right)$ drive a finite-state machine that segments each repetition into lift/hold/lower phases. Five fixed-threshold compensation metrics are aggregated into the quality score *Q* and shown as incremental WebGL feedback. The entire “frame → pose → metrics → feedback” loop runs locally; the raw video never leaves the device, and only derived data (keypoints and metrics) may be exported by the user.

The proposed system is a single client-side loop that converts frontal monocular RGB video into interpretable geometric feedback—expressed in terms clinicians recognize, though its clinical effectiveness is not yet established—without any server-side computation (Figure 1). A seated participant faces an ordinary webcam, and the browser captures and processes the video stream through WebRTC so that no raw image leaves the device. Each frame is passed to BlazePose (MediaPipe Pose), which regresses 33 skeletal landmarks together with per-landmark visibility scores (Section 3.2). The landmark coordinates are normalized by frame size and exponentially smoothed (Section 3.3); from the stabilized skeleton, the arm-elevation angle Θ(t), the elbow angle $\theta_{\mathrm{elb}}\left(t\right)$, and the associated amplitude and velocity features are computed. The temporal derivatives of Θ(t) feed a finite-state machine that segments each repetition into *lift*, *hold*, and *lower* phases (Section 3.4). Over the active interval, five normalized compensation metrics (designed to limit body-size dependence) are evaluated against empirically calibrated preliminary thresholds and condensed into a single aggregate quality score *Q* (Section 3.5). Results are rendered locally as incremental WebGL feedback—trajectory plots, a phase-distribution histogram, and a left/right elbow comparison (Section 3.7). The choice of a seated shoulder abduction to roughly $90^{\circ }$ in the frontal plane is deliberate: because the movement unfolds in a plane parallel to the image sensor, the dominant arm trajectory and the abduction angle are preserved under projection with negligible foreshortening so that all metrics can be computed directly from the two-dimensional image-plane coordinates without monocular depth estimation.

### 3.2. Backbone Selection

The pose backbone was selected under two hard constraints imposed by the target deployment: reliable localization of the anatomical references the five metrics need and real-time inference on commodity laptops *without* a discrete GPU. We compared OpenPose, PoseNet, MoveNet, and the BlazePose family on both accuracy and on-device efficiency (Table 2). We note first that the required landmarks are *not* what separates the candidates: the shoulder–elbow–wrist axis of the elbow angle, the ear points for head tilt, and the shoulder-to-hip midline for trunk lean are all present in the COCO seventeen-keypoint skeleton that PoseNet and MoveNet output, so those backbones are not excluded by missing anatomy. The deciding factors are instead implementation-level. BlazePose returns 33 landmarks, which is a denser topology than the metrics strictly require but one that keeps the shoulder-girdle and ear landmarks well separated; it tracks across frames rather than detecting each frame independently, which stabilizes the shoulder line during the partial self-occlusion typical of a seated participant; it adds a mature in-browser runtime (its optional depth channel is not used here); and among the candidates, PoseNet is also the least accurate. These properties, not the landmark set, drive the choice. We do *not* rely on the per-landmark visibility score to gate invalid points: as Section 3.3 explains, that score stays near-maximal exactly when a landmark leaves the frame, so gating is done on image position instead. The converse case—a landmark inside the frame but poorly localized—is not detected by either signal: no confidence threshold is applied, and such a point contributes to the metric as if it were reliable. Smoothing limits the damage to single-frame excursions, and the 250 ms persistence rule prevents an isolated bad frame from raising a flag, but a sustained localization error would propagate. Finally, BlazePose’s direct coordinate regression keeps per-frame latency constant regardless of how many people are in the frame.

Among the remaining real-time candidates, we adopted the *Full* BlazePose variant as the best accuracy–efficiency compromise within the no-GPU device class. OpenPose was excluded because sustained 30 FPS operation requires a discrete accelerator; on laptop CPUs, its throughput barely reaches 10 FPS (9.8, Table 2), useful only as an offline reference rather than as the core of an interactive service. The heavier BlazePose Heavy variant was excluded for the same reason. Although it improves mAP, its reported accuracy on the large shoulder-girdle joints our metrics depend on is close to that of Full, while on integrated graphics, its frame rate falls sharply; it is therefore appropriate only when a discrete GPU is available. The lighter BlazePose Lite is faster still but sacrifices roughly fifteen mAP points, degrading localization of the very joints the metrics measure. The *Full* variant retains the dense 33-landmark topology at an 81 FPS single-model throughput on a laptop with integrated graphics, well above the ≥25 FPS real-time target, which corresponds to a per-frame budget of about 12 ms—comfortably inside the 40 ms budget for responsive visual feedback. We emphasize that these inference figures characterize the backbone in isolation; the end-to-end pipeline rate on deployment hardware, which additionally includes capture, normalization, metric computation, and rendering, is the 22–30 FPS reported in Section 4.5.

### 3.3. Feature Extraction

All features are derived geometrically from the smoothed landmark stream so that the descriptors are invariant to camera resolution and viewing distance and are designed to limit dependence on participant body size. These properties are intended to make the metrics in Section 3.5 more comparable across subjects and acquisition setups, although cross-morphology comparability of the angular and baseline-referenced metrics is not empirically tested here.

The pose estimator returns 33 body landmarks per frame. Each landmark is a two-dimensional point

(1) $$p_{i}=\left(x_{i},y_{i}\right),$$

in image coordinates with the *x*-axis pointing right and the *y*-axis pointing down. The metrics use the ears (7 left, 8 right), shoulders (11, 12), elbows (13, 14), wrists (15, 16), and hips (23, 24). The angle between two vectors is denoted

(2) $$∠\left(u,v\right)=\arccos\;\left(\frac{u\cdot v}{\left\|u\|\;\|v\right\|}\right).$$

Raw pixel coordinates depend on the sensor resolution, so they are normalized to a resolution-independent frame. Crucially, the two image axes are normalized by the *same* scale—the frame height *H*—rather than by *W* and *H* separately, so that the image aspect ratio is preserved and angles measured in the image plane are not distorted:

(3) $$x_{i}^{\prime }=\frac{x_{i}}{H},\;y_{i}^{\prime }=\frac{y_{i}}{H},\;y_{i}^{\prime }\in \left[0,1\right],\;\;x_{i}^{\prime }\in \left[0,\;W/H\right].$$

Normalizing each axis by its own dimension (x/W,y/H) would apply different horizontal and vertical scale factors whenever W≠H, which does not preserve vector directions and would make every angle and the shoulder-elevation ratio depend on the capture aspect ratio; the isotropic convention above avoids this. To quantify what is at stake, we took the recorded landmarks and re-normalized them as the estimator would have done had the same scene been captured at other aspect ratios; then, we recomputed the metrics under both conventions (Table A1). Under the per-axis normalization, the metrics vary by up to 24–$36^{\circ }$ across aspect ratios (and are zero only at 1:1, where W=H removes the anisotropy), whereas under the isotropic normalization, the deviation is at the floating-point level. We are explicit about what this establishes: it is an analytical re-projection of one recorded dataset, demonstrating that the corrected formulation is invariant to the frame aspect ratio *by construction* rather than an empirical comparison of physically different cameras. Effects that accompany a genuine change of capture device—different optics, sensor noise, rolling shutter, or lens distortion—are not addressed by this check and remain untested (Section 5).

When a landmark leaves the field of view, the estimator extrapolates its position and still reports a near-maximal visibility score (close to 1.0); because the visibility score is thus unreliable precisely when the landmark is invalid, we do not threshold on it and instead gate on image position. Frames in which any reference landmark falls outside the image bounds ($x_{i}^{\prime }\in \left[0,W/H\right]$, $y_{i}^{\prime }\in \left[0,1\right]$) are excluded from that metric and from calibration; only in-frame frames contribute. In practice, this exclusion is negligible for the seated frontal task: the reference landmarks (nose, shoulders, and hips) remained within the frame in over 99.9% of the 28,611 frames that fall inside the 621 analyzed repetitions of the detection sample (Section 4.1). No repetition was excluded on this ground and none lost its resting baseline, so all 621 repetitions entered the analysis and frame gating introduces no material selection bias.

Pose estimators exhibit frame-to-frame coordinate jitter, and differentiation greatly amplifies it; without smoothing, the velocity and acceleration that drive phase segmentation would be dominated by noise. Each landmark is therefore stabilized with a causal exponential moving average before any derivative is computed:

(4) $${\tilde{p}}_{i}\left(t\right)=\alpha \;p_{i}\left(t\right)+\left(1-\alpha \right)\;{\tilde{p}}_{i}\left(t-1\right),\;\alpha \in \left(0,1\right).\;$$

The smoothing coefficient α trades responsiveness against noise suppression: values near one track fast motion with little lag, whereas smaller values yield stronger denoising. We set α=0.35, which at 30 FPS corresponds to a first-order cutoff of ≈2.1 Hz—above the ≲1 Hz bandwidth of the rehabilitation movements, so the motion of interest is preserved—while still reducing frame-to-frame landmark jitter by about 20% (measured on the resting-posture frames of the validation cohort) at a group delay of only ≈1.9 frames (≈62 ms). Tightening to α=0.2 would cut a further margin of jitter but double the lag to ≈133 ms, which is past the feedback budget; α=0.35 is the balance adopted.

The progress of a repetition is tracked by the arm-elevation angle—the angle of the upper arm to the downward image vertical $e_{y}=\left(0,1\right)$—computed for each side s∈{L,R}:

(5) $$\Theta_{s}\left(t\right)=∠\;\left(p_{\mathrm{elbow}}^{s}-p_{\mathrm{shoulder}}^{s},\;e_{y}\right).$$

Measuring elevation against the image vertical, rather than against the trunk axis, keeps it independent of trunk lean so that a leaning torso does not inflate the apparent arm angle. At rest, the arm hangs down and $\Theta_{s}\;\approx \;0$; at full lateral elevation, $\Theta_{s}\;\approx \;90^{\circ }$. For an upright seated participant, the image vertical coincides with the trunk axis, so this angle matches the abduction angle compared against the synchronized IMU reference in Section 4. The segmentation signal Θ(t) is the elevation of the moving arm, and its first and second temporal derivatives drive the finite-state machine,

(6) $$\dot{\Theta }=\frac{d\Theta }{dt},\;\ddot{\Theta }=\frac{d^{2}\Theta }{dt^{2}},$$

Because the delivered frame rate varies (22–30 FPS, Section 4.5), these derivatives are formed from the measured inter-frame interval rather than from a nominal frame period: each frame carries its own timestamp, the elapsed time Δt between consecutive processed frames is computed from those timestamps (clamped at 100 ms so that a dropped detection cannot inflate it), and velocities are expressed per second. The violation window of Section 3.5 is likewise accumulated in milliseconds of elapsed time, not in frame counts, so a repetition is judged identically at 22 and at 30 FPS. Here, the velocity $\dot{\Theta }$ separates active motion from rest and the acceleration $\ddot{\Theta }$ marks the turning point between raising and lowering the arm.

The elbow angle quantifies arm straightness as the angle at the elbow between the upper-arm and forearm vectors, used by the first compensation metric to detect movement performed through elbow flexion rather than true shoulder abduction:

(7) $$\theta_{\mathrm{elb}}^{s}=∠\;\left(p_{\mathrm{shoulder}}^{s}-p_{\mathrm{elbow}}^{s},\;p_{\mathrm{wrist}}^{s}-p_{\mathrm{elbow}}^{s}\right),\;s\in \left\{L,R\right\}.$$

A straight arm gives $\theta_{\mathrm{elb}}\;\approx \;180^{\circ }$; bending the elbow lowers it. Note that Θ (arm-to-vertical, the raise of the whole arm) and $\theta_{\mathrm{elb}}$ (the angle at the elbow) are distinct quantities: a straight arm held at rest has $\theta_{\mathrm{elb}}\;\approx \;180^{\circ }$ yet Θ≈0.

### 3.4. Phase Segmentation

Because compensation criteria are only meaningful while the arm is actually moving, each repetition is segmented into kinematic phases by a finite-state machine (FSM) driven by the arm-elevation angle Θ(t) and its velocity $\dot{\Theta }\left(t\right)$ against fixed thresholds. The FSM has four states: *rest*, *lift*, *hold*, and *lower*. In *rest*, the angle stays close to its baseline $\Theta (t_{0})$ and no criteria are evaluated. The machine enters *lift* when the arm-elevation angle first reaches an initiation threshold of $25^{\circ }$, which also marks the repetition start $t_{s}$. It moves to *hold* once the arm passes $55^{\circ }$ of elevation and the angular speed falls below a fixed threshold $\omega_{\mathrm{th}}=20^{\circ }/s$; downward motion faster than $\omega_{\mathrm{th}}$ switches the machine to *lower*. When the elevation falls back below $18^{\circ }$, the machine fixes the repetition end $t_{e}$ and reverts to *rest*. All criteria are checked only over the resulting active interval $T=\{t_{s},\ldots ,t_{e}\}$. These fixed geometric thresholds (Table A2) keep phase switching reproducible across participants without any per-subject retraining.

The state boundaries are anchored to a calibrated functional zero rather than to absolute image coordinates. Because the first seconds of a recording are often unsettled (the participant may still be sitting down), the baseline is taken as the *median* over the in-frame rest-phase frames—when the arms hang down—rather than from a fixed initial window; the median is robust to a noisy start. To make this well defined for a live causal pipeline, the functional zero is seeded from an initial low-velocity settling window and then refined over the rest-phase frames the FSM subsequently confirms, so a baseline exists before the first repetition is segmented. The same rest-phase frames fix the references used by the displacement metrics of Section 3.5: the resting shoulder height ${\bar{y}}_{\mathrm{sh},0}=\mathrm{med}\;\frac{1}{2}\left(y_{11}+y_{12}\right)$, the shoulder width $w_{\mathrm{sh},0}=\mathrm{med}\;\left\|p_{11}-p_{12}\right\|$, and the resting ear-line tilt ${\mathrm{earTilt}}_{0}$.

### 3.5. Compensation Metrics

**Figure 2:**
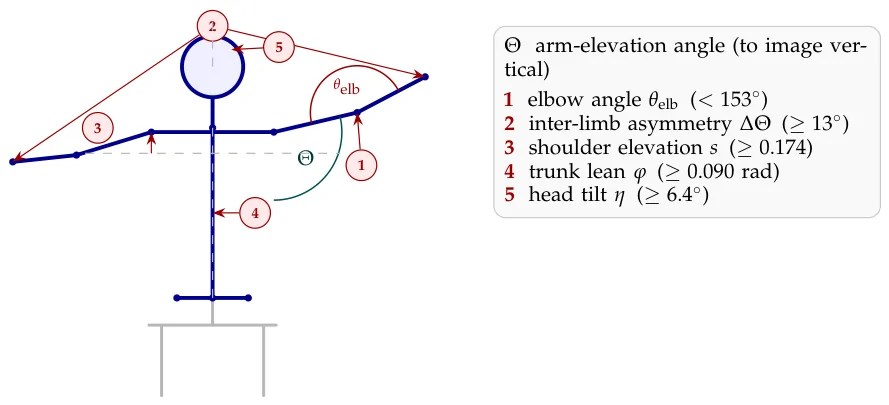
The five compensation metrics on a seated, frontal-view skeleton. Θ is the primary shoulder abduction angle (Equation (5)); the numbered markers locate each fixed-threshold metric of Table 3: (1) incomplete elbow extension, (2) inter-limb asymmetry of the arm-elevation angles, (3) shoulder elevation relative to the resting baseline (dashed), normalized by shoulder width, (4) lateral trunk lean from the vertical (dashed), and (5) head tilt measured as ear-line inclination.

From the segmented kinematics, we derive five normalized geometric metrics that together cover the compensatory patterns most frequently observed in upper-limb rehabilitation of the shoulder girdle: substituting elbow flexion for shoulder abduction, inter-limb asymmetry, compensatory shoulder elevation, trunk lean, and head tilt. Each metric is defined on the normalized, image-plane coordinates of Section 3.3, which keeps it invariant to resolution and distance and limits its dependence on body size. Each is then compared against a fixed threshold so that the detector requires no per-subject tuning. Displacement-based metrics are referenced to the calibrated functional zero (the baseline landmark positions $t_{0}$) and normalized by the resting shoulder width, while angular metrics use the same vector formulation as the primary angles. Figure 2 shows the five metrics on a seated frontal skeleton together with the primary arm-elevation angle Θ.

1. **Incomplete elbow extension.** The minimum elbow angle of the two arms, $\theta_{\mathrm{elb}}^{\min}=\min\left(\theta_{\mathrm{elb}}^{L},\theta_{\mathrm{elb}}^{R}\right)$ from Equation (7), flags movement performed through elbow flexion rather than true shoulder abduction. It is flagged when $\theta_{\mathrm{elb}}^{\min}<153.4^{\circ }$.
2. **Inter-limb asymmetry.** The absolute difference of the two arm-elevation angles (Equation (5)), $\Delta \Theta =|\Theta_{L}-\Theta_{R}|$. Because elevation is measured against the image vertical, trunk lean does not contaminate it. It is flagged when $\Delta \Theta \geq 13.2^{\circ }$ but only while the trunk is upright (φ<0.072 rad) and both arms are extended ($\theta_{\mathrm{elb}}^{\min}\geq 153.4^{\circ }$); this suppression keeps a lean or an elbow bend from being misread as asymmetry. Because the suppression is applied per frame, a repetition that simulates elbow flexion can still accrue an asymmetry flag on the frames where both arms are momentarily extended, which is one contributor to the asymmetry firing seen on elbow-flexion trials in the validation (Section 4.4); a genuine co-occurring asymmetry, which the annotation protocol permitted and labeled, is another.
3. **Shoulder elevation (shrug).** The upward shift of the shoulder line from its resting height is normalized by shoulder width so that it is scale-independent:
  (8) $$s=\max\;\left(0,\;\frac{{\bar{y}}_{\mathrm{sh},0}-{\bar{y}}_{\mathrm{sh}}}{w_{\mathrm{sh},0}}\right),\;{\bar{y}}_{\mathrm{sh}}=\frac{1}{2}\left(y_{11}+y_{12}\right).$$
  It is flagged when s≥0.174. A geometric confound is nonetheless built into Equation (8): during normal abduction, the glenohumeral joint center rises and, in the frontal projection, the acromion travels upward with the arm even without a true shrug, so ${\bar{y}}_{\mathrm{sh}}$ decreases (the shoulder line rises) as a by-product of the movement being assessed. Because *s* references the *mean* shoulder height against a single resting baseline, it cannot separate this projection-coupled rise from a genuine scapular elevation; as the validation shows (Section 4.4), residual shoulder displacement from coupled compensations still raises this flag. Referencing one shoulder to the contralateral side, gating on the movement phase, or adding a depth or inertial channel could disentangle the two (Section 5), and until then, the metric is treated as research-grade.
4. **Trunk lean.** The angle of the trunk axis to the upward vertical,
  (9) $$\varphi =∠\;\left(p_{\mathrm{sho}}^{\mathrm{mid}}-p_{\mathrm{hip}}^{\mathrm{mid}},\;\left(0,-1\right)\right),$$
  where $p^{\mathrm{mid}}$ is the midpoint of the left and right landmarks; this is flagged when φ≥0.090 rad.
5. **Head tilt.** The change in ear-line inclination from its resting value, $\eta =|\mathrm{earTilt}-{\mathrm{earTilt}}_{0}|$ with $\mathrm{earTilt}=∠\;\left(p_{7}-p_{8},\;\left(1,0\right)\right)$ (the inclination of the line joining the ears to the horizontal). It is flagged when $\eta \geq 6.4^{\circ }$, and it is raised only while the trunk is upright (φ<0.072 rad), since a trunk lean also tilts the ear line.

Each metric $M_{j}$ raises a per-frame flag $f_{j}\left(t\right)$ when its criterion is met. A repetition is flagged for metric *j* once the cumulative in-frame time with $f_{j}=1$ over the active phases (lift/hold/lower) reaches 250 ms (≈6–8 frames at the 22–30 FPS of Section 4.5). The window was fixed a priori rather than optimized against a criterion, and it is bounded from both sides: it must exceed the group delay of the smoothing filter (≈62 ms) and span several frame intervals so that an isolated mis-localization cannot raise a flag, while remaining short against the median repetition duration of 1.4 s, so that a sustained deviation is caught within the movement rather than after it; these per-repetition flags are the output validated against clinician annotation (Section 4). For a compact summary of execution quality, the same per-frame flags are pooled into an aggregate score

(10) $$Q=1-\frac{1}{n\;T}\sum_{j=1}^{n}\sum_{t=1}^{T}f_{j}\left(t\right),$$

the fraction of compensation-free metric-frames over the *T* in-frame active frames (*n* metrics), ranging from 0 to 1. *Q* is an unvalidated, equal-weighted convenience summary: only its constituent per-repetition flags are validated (Section 4), so *Q* inherits their reliability, including the weaker shoulder-elevation flag.

#### Threshold Calibration

The thresholds in Table 3 were calibrated on a separate calibration cohort of nine healthy participants (disjoint from the eighteen used for validation), performing the same six conditions—one compensation-free and one per target compensation—for 292 repetitions in total (80 correct, 39 elbow, 45 asymmetry, 44 shoulder, 39 trunk, 45 head). Two participants did not complete the full set: one contributed no trunk-lean block and one neither an elbow-flexion nor a shoulder-elevation block, so the elbow, shoulder, and trunk operating points rest on eight of the nine participants and the remaining three rest on all nine. For each metric, the operating point was chosen on this cohort to separate the compensation-free condition from the targeted compensation by Youden’s index on the ROC curve, subject to a false-positive rate on correct execution of at most 10%; the metrics are well separated on the calibration cohort. Computed on the per-repetition statistic the detector itself thresholds—the most extreme value sustained for the accumulation window—the area under the ROC curve separating each targeted compensation from the compensation-free condition is 1.00 for incomplete elbow extension, 0.99 for inter-limb asymmetry, 0.96 for shoulder elevation, 1.00 for trunk lean, and 1.00 for head tilt. The same optimization criterion and the same false-positive constraint were applied to all five. These values are reproducible from the archived repetition-level tables. The calibration sessions were run by a clinician, who instructed and demonstrated each condition as in the detection study, but no clinician rated the calibration repetitions and no threshold was chosen or reviewed by a clinician. Note that this calibration uses the *instructed* condition of each calibration repetition as its reference rather than a per-repetition clinician label; since participants were free to produce co-occurring compensations (Section 4.4), a small number of calibration repetitions may carry an unlabeled additional sign, which makes the reported separation a mild over-estimate of what a label-based calibration would show. The thresholds were then frozen and evaluated on the eighteen validation participants against clinician labels so that no threshold is tuned on its own validation data. The threshold *values* are thus data-driven—set by operating-point selection on the calibration set rather than taken from published clinical cut-offs—whereas the *choice* of which geometric signs to measure is motivated by the compensatory patterns reported in the rehabilitation literature. The violation window (250 ms) and the trunk-suppression threshold were set on the same calibration cohort and were not varied in the validation. The suppression threshold sits at four fifths of the trunk-lean operating point (0.072 against 0.090 rad), so that a developing lean gates the asymmetry and head metrics before it is itself flagged, and it was rescaled in that same proportion when the coordinate convention was corrected; the 250 ms window, chosen to reject excursions shorter than a quarter second, was not part of the five-threshold sensitivity sweep of Section 4.6. The metrics remain interpretable throughout: each is an explicit geometric quantity compared against a stated threshold rather than a black-box score.

### 3.6. Statistical Analysis

The three samples of healthy volunteers—nine for threshold calibration, eighteen for compensation detection, and ten for the IMU angle validation, 37 participants in total—are *disjoint* with no individual appearing in more than one (Section 4.1). Consequently, no threshold is evaluated on the data that set it, and the angular validation and the detector validation rest on independent samples.

Both validation studies are small by design—a feasibility and metrological evaluation on healthy volunteers rather than a clinical-efficacy trial—so no a priori power calculation was performed; instead, every reported detector performance estimate is accompanied by an uncertainty interval computed with the participant as the unit of analysis so that the correlation among repetitions of the same person is not mistaken for independent evidence; the inertial-agreement figures are reported as per-subject summaries with their across-subject spread.

For the compensation detector, all 95% confidence intervals (per-class precision, recall/sensitivity, specificity, false-positive rate, $F_{1}$, and the macro-average) are obtained by a cluster bootstrap that resamples the 18 participants with replacement over 10,000 iterations and recomputes the statistic on each resample; because whole participants are resampled, the 621 repetitions are never treated as 621 independent observations. This follows the established treatment of agreement statistics on clustered responses, where a cluster-level bootstrap has been shown to estimate the standard error of κ more accurately, and to give better interval coverage, than an asymptotic estimate that ignores within-cluster dependence [50]. The same participant-stratified procedure and seed are used for every detection interval reported. Cohen’s κ is used for inter-rater and system-to-rater agreement, and the whole-set, per-clinician evaluation (Section 4.4) is taken as the primary result with the consensus subset reported only as an upper bound.

For the IMU validation, the per-frame error was aggregated by the two-level scheme described in Section 4.2: a per-subject median and interquartile range over that subject’s five repetitions, then a group median [Q1–Q3] over the ten subjects, so that no summary statistic treats the five cycles of one participant as five independent observations. We note one limitation of this scheme: it summarizes *within* subjects before aggregating, but it is not a formal repeated-measures Bland–Altman analysis [51,52], which would model the within-subject and between-subject variance components explicitly and would in general yield limits of agreement different from those obtained by either pooling frames or taking medians of per-subject limits. A repeated-measures re-analysis of the inertial data is therefore left as future work, and the limits reported here should be read as descriptive.

### 3.7. On-Device Implementation

The pipeline is implemented entirely as a client-side browser application so that the complete “frame → pose → metrics → feedback” loop executes on the patient’s own machine. Pose estimation uses MediaPipe Tasks Vision (v0.10.14; Google LLC, Mountain View, CA, USA) with the pose_landmarker_full model (float16), running in VIDEO mode with numPoses=1 and the GPU (WebGL) delegate over the WebAssembly runtime; the smoothing, segmentation, and metric layers are plain JavaScript. Video is captured and handled through WebRTC with all frame processing performed locally in the browser. The estimator runs on the end device; the WebAssembly runtime and the model weights are fetched once from a public CDN, after which no image data is transmitted (the raw video never leaves the device). Both shoulders and both arms are required to be in frame throughout, since inter-limb asymmetry and shoulder elevation are bilateral by construction; a unilateral setup or a missing contralateral arm therefore falls outside the current scope and is noted as a limitation. The user interface overlays the recovered skeleton on the live video and signals execution correctness in place: when a compensation is detected, a corrective notification is raised with a computational latency below 120 ms—the total delay from the onset of a compensation is larger, since a violation must persist for the 250 ms window (Section 4.5)—which is fast enough for the participant to adjust the movement within the same repetition.

Visual feedback is composed in a single WebGL layer that updates incrementally as the exercise proceeds, rendering three views: a trajectory plot of the abduction angle Θ(t) and amplitude d(t) that grows one sample at a time; a bar histogram of the phase distribution, reporting the lift/hold/lower share of the cycle as $P_{\mathrm{phase}}=\left(t_{\mathrm{phase}}/T_{\mathrm{cycle}}\right)\times 100\%$ together with its mean, minimum, and maximum; and a comparative plot of left and right elbow flexion–extension over normalized cycle time with the right limb drawn in purple and the left in turquoise.

#### Data Handling: Two Distinct Configurations

Because privacy claims are easy to overstate, we separate the patient-facing pipeline from the instrument used to collect the present dataset; the two deliberately handle data differently.

*(i) The deployed pipeline.* In normal use, no image ever leaves the machine: frames are drawn from the camera, passed to the estimator, and discarded, and neither video nor keypoints are transmitted to any server. Derived data (per-frame keypoints and the aggregate metrics) exist only in the page session and may be exported to CSV by an explicit, user-initiated action; the exported file stays on the user’s disk unless the user chooses to share it. The client contains no telemetry, analytics, or usage reporting. There is, however, one external dependency to declare: the estimator runtime and the model weights are fetched once over HTTPS from a public content delivery network before inference starts. Those requests download code and weights and carry no image, keypoint, or session data, but they do disclose to the CDN that a client requested the model, which is the residual network footprint of an otherwise offline pipeline. Camera access is requested through the standard browser permission prompt and is revocable by the user at any time.

*(ii) The research instrument used for this study.* Blinded clinician annotation requires clinicians to watch the movement, so the acquisition tool was built to record: the video and the per-frame keypoints of each block were uploaded to a study server on the laboratory network, where they remain, and served back to the annotation interface. Access is restricted by a per-rater token. Recordings are stored under pseudonymous participant codes (for example P07); the key linking a code to an identity is kept separately and is available only to the responsible investigator. Every participant gave separate written consent to the video recording itself, to the processing of the video as biometric personal data, and to the use of anonymized results in publications, and they also retain the right to withdraw consent and to require deletion of their data; no video frames are reproduced in this paper. This recording configuration is a property of the *study*, not of the deployed system, and none of the on-device claims above depend on it.

Two caveats apply to both configurations. Derived keypoints are not innocuous: a landmark stream is health-related and, in principle, biometric, so “the video never leaves the device” bounds the raw image stream and is not a claim that the exported data are free of privacy implications. And the local CSV export is written unencrypted, so protection of exported files rests on the security of the user’s own machine (Section 5).

The client meets the real-time budget that interactive feedback demands. On commodity consumer devices, the end-to-end pipeline sustains 22–30 FPS—mid-range laptops reach 22–25 FPS, while modern configurations such as the Apple M1 and the Intel i5-12500H hold 27–30 FPS—at a moderate CPU load of 9–20%, as detailed in Section 4.5; these are complete-pipeline rates that are distinct from the backbone-only inference throughput of Table 2. Five of the six tested configurations reach the ≥25 FPS target for responsive visual feedback; the remaining one sustains 22 FPS. We did not test smartphone or tablet hardware end-to-end, so we make no mobile real-time claim; the reported rates apply to the six tested laptop/browser configurations. Because the implementation depends only on a browser, a webcam, and integrated graphics, it requires neither motion-capture markers nor a discrete GPU, which makes the system practical for unsupervised home use on the tested class of devices.

## 4. Experiments and Results

### 4.1. Participants

All recordings were made on healthy adult volunteers. Three *disjoint* samples were formed, and no participant took part in more than one of them, so that the calibration, angular-validation, and detection results are obtained on independent people (Table 4). A first sample (N=10) served the accuracy check of the markerless shoulder-abduction angle against a synchronized inertial reference (Section 4.2); a second (N=9) was used exclusively to calibrate and then freeze the metric thresholds (Section 3.5); a third (N=18) was used to evaluate compensation detection against the independent blinded annotation of two clinicians (Section 4.4). The total number of unique participants was therefore 37. Across the three samples, participants were aged 24.0±4.3 years (mean ± SD; median 24, range 18–32), spanned roughly 165–190 cm in stature and 60–120 kg in body mass, and approximately 75% were male; the sex ratio was close to this value in each of the three samples separately. We report stature and mass as approximate ranges because anthropometry was not systematically measured: the study protocol recorded neither height, mass, nor limb lengths per participant, and handedness was not recorded, either. This has a direct consequence for the metric design—the claim that the normalized metrics limit body-size dependence rests on their construction (angles and shoulder-width ratios) and cannot be checked empirically against measured body dimensions on this dataset (Section 5).

All three samples were recorded under the same acquisition setup: the participant seated facing a frontal RGB webcam at approximately eye level, 2–2.5 m away, in ordinary indoor lighting, with the camera requested at 1280×720 and 30 FPS and the stream processed locally on the recording machine. No variation of camera height, distance, viewing angle, or lighting was introduced, so the dataset characterizes one controlled configuration rather than a range of home conditions.

Volunteers were included if they had no diagnosed neurological disease, no marked orthopedic pathology of the shoulder girdle, spine, or upper limbs, no injury to the tested limb, and no pain that would limit the motor task; they had to be able to follow the instructions, to remain seated for the duration of a recording block, and to perform frontal-plane shoulder abduction safely. Recordings were excluded from analysis if the motor task was not completed as specified or if the recording was technically unusable. All participants gave written informed consent to take part and to the processing of the resulting data. We note that this is a young, predominantly male, healthy sample; it is not representative of the post-stroke population the system ultimately targets, and the limitations of generalizing from it are discussed in Section 5.

### 4.2. IMU Validation Protocol

**Figure 3:**
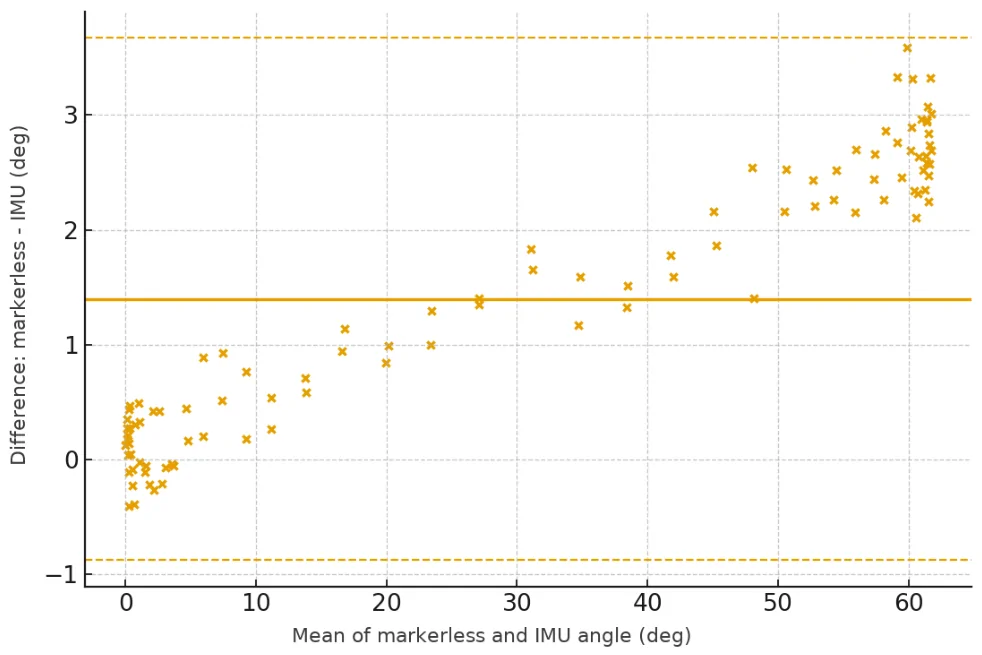
Bland–Altman representation of the markerless versus inertial abduction angle. Each point is one percentage of the time-normalized movement cycle, averaged across the ten participants; the solid line is the mean difference over those points ($1.40^{\circ }$) and the dashed lines the corresponding limits of agreement ($-0.87^{\circ }$ and $+3.68^{\circ }$). Because averaging across participants removes between-subject scatter, these limits describe the agreement of the *mean* trajectory and are narrower than limits for individual measurements; the per-subject limits are those summarized in Table 5. The upward trend of the differences with the mean angle is the proportional bias discussed in the text.

The accuracy of the markerless abduction angle Θ(t) of Equation (5) was validated against a synchronized inertial reference. One point of provenance matters here. This angular study was conducted earlier and separately from the compensation-detection study, on its own processing path, in which the image-plane angle was computed with a single common scale for both axes—that is, in the isotropic convention of Section 3.3. The anisotropic per-axis normalization that had to be corrected in the compensation detector was therefore never applied here, and the agreement statistics below are unaffected by that correction; they were not, and could not be, recomputed, because the raw inertial recordings are no longer retained. Ten participants (N=10) each performed five repetitions of the standardized motor task—a seated shoulder abduction in the frontal plane, instructed toward approximately $90^{\circ }$—yielding fifty time-normalized cycles (10×5). In execution, the participants did not raise the arm to the horizontal: the group-averaged trajectory plateaus at about $61^{\circ }$ (Figure 3), so the angular validation covers roughly the 0–$62^{\circ }$ interval and does not extend to the full anatomical abduction range. The reference angle was obtained from two wireless inertial measurement units of a STEDIS Kinematics system (Neurosoft, Ivanovo, Russia) sampling at 200 Hz, each combining a tri-axial accelerometer and a tri-axial gyroscope: one unit was fixed to the sternum, defining the trunk as the base segment with its axes aligned to the torso, and the second was fixed to the lateral surface of the mid-humerus of the tested arm, defining the working segment. After a brief sensor-to-segment calibration, the reference abduction angle $\theta_{\mathrm{IMU}}\left(t\right)$ was computed from the segment quaternions by rotating the longitudinal humerus axis into the global frame, projecting it onto the trunk plane to remove the anteroposterior component, and taking the angle between this projection and the trunk vertical with the sign carried by the lateral component. By construction, it reads zero at the neutral posture and increases with lateral elevation, so the inertial reference and the image-plane estimate describe the same frontal-plane abduction. Because the inertial reference is itself projected onto the frontal plane, the agreement quantifies in-plane abduction accuracy and does not certify the capture of out-of-plane motion. The video channel was the common acquisition setup of Section 4.1 with the stream processed locally by the client module on the participant’s machine and no remote computation.

The two signals were brought into correspondence before any error metric was computed. A functional zero was established by averaging each channel in the neutral anatomical posture and subtracting the resulting offset, after which both signals were filtered with a zero-phase second-order low-pass filter (cutoff 4–6 Hz). Temporal alignment was performed by maximizing the cross-correlation of the angle *derivatives*, and the estimated lag was corrected. Because the inertial channel is sampled at 200 Hz and the video channel at 30 FPS, both were then resampled to a common 30 Hz grid—the rate of the slower channel so that the comparison is made at the temporal resolution the markerless method actually delivers—and time-normalized to 0–100% of the cycle with lift/hold/lower phase labels. Synchronization between the video and inertial channels was performed in software rather than by a hardware trigger, which is why the residual lag had to be estimated from the signals themselves. Per-frame error was defined as $e_{k}=\theta_{\mathrm{HPE}}\left(t_{k}\right)-\theta_{\mathrm{IMU}}\left(t_{k}\right)$, summarized by the mean absolute error (MAE) and root-mean-square error (RMSE), and agreement was assessed by Bland–Altman analysis (bias and limits of agreement, LoA) [51] and Lin’s concordance correlation coefficient (CCC) with a 95% confidence interval; CCC was preferred over the Pearson coefficient because a high linear correlation can mask a systematic offset. Group values were obtained by a two-level scheme: a per-subject median and interquartile range over the five repetitions, followed by a group median [Q1–Q3] over the ten subjects. All acceptance criteria were *pre-specified*—fixed before the analysis—namely MAE $\leq 5^{\circ }$, $\left|\mathrm{bias}\right|\leq 2^{\circ }$, and LoA width $\leq 10^{\circ }$; these bounds were specified for a working range of 10–$90^{\circ }$, whereas the range actually exercised was 0–$62^{\circ }$, so the criteria are evaluated only over the interval the data cover. An auxiliary Pearson r≥0.90 was also specified. Conformity with these bounds is assessed *descriptively*: a criterion is met when the per-subject estimate and the across-subject spread of the per-subject values both lie inside the bound. We deliberately do not present this as a formal equivalence test, because the bracketed ranges are interquartile ranges over the ten subjects rather than confidence intervals, and the per-frame data needed to construct inferential intervals are no longer available.

Two of these preprocessing steps deserve explicit comment, because they are *not* available to the deployed system. The zero-phase filter and the cross-correlation realignment are offline operations: the first uses future samples, and the second removes a residual timing offset after the fact. The live pipeline is strictly causal—a single exponential moving average with a group delay of about 62 ms (Section 3.3) and no lookahead. The agreement reported below therefore characterizes the accuracy of the *geometric estimator* with timing effects removed, and it is an optimistic estimate of what the live, latency-inclusive system delivers instant by instant. The gap is concentrated in the transient phases: a fixed delay τ maps to an instantaneous angular error of roughly $\tau \;\dot{\Theta }$, so at the angular velocities of this movement’s lift and lower phases, the causal path would carry a few additional degrees of transient error, while the *hold* plateau—which determines the amplitude read-out—is essentially unaffected because $\dot{\Theta }\approx 0$ there. The phase-stratified results below are consistent with this reading. We did not measure the causal pipeline against the inertial reference directly, and the raw recordings needed to do so retrospectively are no longer available; a causal-only comparison is left as required future work.

### 4.3. Agreement with the IMU Reference

The markerless estimate met every pre-specified criterion. The group trajectory error (Table 5) was a median MAE of $2.18^{\circ }$, well below the $5^{\circ }$ tolerance, with an RMSE of $2.61^{\circ }$, indicating few large outliers. The median per-subject bias of $0.92^{\circ }$ stays within the $\pm 2^{\circ }$ bound, and the limits of agreement span $7.35^{\circ }$, which is within the pre-specified $10^{\circ }$ ceiling over the 0–$62^{\circ }$ range the recordings cover. How that agreement is distributed across the range is examined below. Lin’s CCC of 0.956 [0.942–0.967] reflects high concordance, capturing both linear correlation and the absence of a systematic shift in a single index. The auxiliary Pearson criterion is met a fortiori: because the CCC is the product of the Pearson *r* and a bias-correction factor no greater than one, r≥CCC=0.956≥0.90.

The phase-stratified breakdown (Table A3) localizes where the residual error arises. Agreement is best during the *hold* plateau (MAE $1.64^{\circ }$), which is the phase that determines the amplitude estimate and is therefore the most clinically load bearing; the transient *lift* and *lower* phases carry slightly larger but still in-tolerance errors (MAE $2.45^{\circ }$ and $2.72^{\circ }$), which we attribute to residual time lag and to the differing filter bandwidths of the two channels during the fastest motion. The interval-based assessment (Table 6) is consistent with this picture, and both conclusions are read descriptively off the per-subject summaries: the across-subject spread of the bias, ${\left[0.38,\;1.47\right]}^{\circ }$, lies entirely inside the $\pm 2^{\circ }$ margin, and that of the MAE, ${\left[1.62,\;2.83\right]}^{\circ }$, lies entirely below the $5^{\circ }$ bound. These are interquartile ranges, not confidence intervals, so the statement is one of conformity rather than of formal statistical equivalence.

These aggregate figures, however, conceal a systematic structure that the Bland–Altman representation makes explicit (Figure 3). The difference is not constant across the range of motion: it is essentially zero at rest and grows with the angle, so the markerless estimate *overestimates* elevation in proportion to its magnitude. Regressing the difference on the mean of the two methods gives a slope of approximately 0.044 degrees per degree ($R^{2}\approx 0.89$)—about a 4.4% scale error—corresponding to a negligible offset near the neutral posture and roughly $+2.7^{\circ }$ at the top of the recorded range. The paper therefore reports a proportional bias rather than its absence, and the constant-bias summary in Table 5 should be read as an average over the trajectory. Practically, this is a well-behaved error: it is systematic and amplitude-proportional rather than random, so it is in principle removable by a scale calibration, but it does mean that at the upper end of the recorded range, the systematic component alone approaches and exceeds the $\pm 2^{\circ }$ margin that the trajectory-averaged bias satisfies. An amplitude-dependent rather than constant error appears to be characteristic of markerless upper-limb estimation: a recent validation against an optoelectronic reference likewise reports its largest deviations at maximum flexion, attributing them to occlusion and joint geometry, although there the sign is reversed and the extreme is underestimated [25].

### 4.4. Compensation Detection vs. Clinician Annotation

The frozen detector was evaluated against expert annotation on a *separate*, held-out cohort, to test whether the five fixed-threshold metrics of Section 3.5 flag the same compensations that clinicians identify by eye. The evaluation set comprises 18 participants who perform, in a balanced design, each of the five target compensations (incomplete elbow extension, inter-limb asymmetry, shoulder elevation, trunk lean, and head tilt) as well as repetitions instructed to be compensation-free, yielding 621 segmented repetitions in total. Throughout, we distinguish the *instructed* condition of a repetition from its *labels*: as described below, a repetition instructed to be compensation-free may still carry a clinician-labeled sign.

The acquisition protocol was clinician-led. Before each condition, a clinician instructed the participant and physically demonstrated both the target movement—a seated frontal-plane abduction to approximately $90^{\circ }$—and the compensation to be simulated so that the amplitude of both the movement and the simulated compensation was standardized by demonstration rather than by an instrumented target. The order of conditions was randomized across participants to avoid systematic fatigue or learning effects. Participants were not required to isolate the instructed compensation: naturally co-occurring compensations were permitted rather than suppressed, since forcing an isolated sign would have produced less realistic movement.

Annotation was carried out by two clinicians who were *not* the clinicians who ran the acquisition sessions, and who had neither observed the recordings being made nor been told which compensation each repetition had been instructed to contain. Each repetition was therefore annotated independently and blind on three counts: to the instructed condition, to the system’s output, and to the other rater’s ratings; the raters assigned the five binary compensation labels from the video alone. Crucially, the raters labeled *every* compensation they observed, not only the one the participant had been instructed to perform. The reference labels therefore describe what is actually visible in each repetition, and off-target flags in Table 7 should not be read as detector errors by construction: a shoulder-elevation flag on an elbow-flexion repetition may correspond to a genuine, clinician-labeled co-occurring sign. This is the correct reference for our purpose—the detector is meant to report the compensations present rather than to recover the experimenter’s instruction—but it does mean the per-condition firing rates mix instructed and spontaneous compensations.

The two raters agreed substantially to almost perfectly with a mean Cohen’s κ of 0.84 across the five metrics and identical labels on all five for 477 of 621 repetitions (76.8%). Per-metric, agreement was highest for trunk lean (κ=0.97), incomplete elbow extension (0.96), and head tilt (0.93), and it was lower for shoulder elevation (0.73) and inter-limb asymmetry (0.62); the reduced agreement on the latter two is itself informative, as these displacement-based signs are intrinsically harder to judge from a single frontal view—even between experts.

Rather than forcing the two raters into a single ground truth, we evaluate the detector in two complementary ways, and we take the whole-set, per-clinician analysis as the primary result. All uncertainty estimates are 95% confidence intervals from a cluster bootstrap that resamples *participants* (not repetitions) with replacement over 10,000 iterations so that the within-participant correlation of repetitions is respected and the intervals are not narrowed by treating the 621 repetitions as independent (Section 3.6).

*Primary analysis—each clinician, all* 621 *repetitions*. The detector is evaluated against *each* rater over the full cohort, the setting closest to real operation, in which ambiguous repetitions are retained rather than removed. The per-clinician macro-$F_{1}$ is 0.75 and 0.72 (95% CI [0.69,0.80] and [0.66,0.78]); per-class point estimates and confidence intervals against each rater are reported in Table 8; the underlying per-clinician confusion counts are given in Table A4. Performance also varies across participants: computed against the consensus reference (the only per-participant breakdown available), the per-participant macro-$F_{1}$ spans 0.38 to 0.96 (median 0.75 over the 18 participants); here, the low tail is driven mainly by shoulder elevation, the weakest sign, on the participants who simulated the subtlest compensations. System-to-clinician agreement is read against clinician-to-clinician agreement: on the well-defined signs, the system agrees with each rater almost as closely as the raters agree with each other (trunk lean and head tilt: system-to-clinician κ=0.84–0.91 versus clinician-to-clinician 0.93–0.97). We state this descriptively: no interval was computed for the difference between these agreement coefficients, so the comparison indicates proximity to human inter-rater variability rather than statistical equivalence to it.

*Secondary analysis—consensus subset, an upper bound.* Against a *consensus reference*—the 477 repetitions on which both clinicians agree—per-class precision, recall, and $F_{1}$ are reported in Table 9, and the full panel of precision, recall (sensitivity), specificity, and false-positive rate with confidence intervals are reported in Table 10 (computed on this subset and therefore subject to the same upper-bound caveat); the detector attains a macro-averaged $F_{1}$ of 0.78 (95% CI [0.72,0.83]) with all five labels correct in 65.6% of repetitions. Because this subset removes the 144 repetitions the clinicians themselves found ambiguous, and those excluded repetitions are precisely those the two raters could not agree on, the consensus subset is an easier evaluation set: the gain over the per-rater estimate is concentrated in the two signs with the lowest inter-rater agreement (inter-limb asymmetry, κ=0.62, and shoulder elevation, κ=0.73). Class prevalence also shifts by up to 0.053 (Table A5). The 0.78 figure should therefore be read as an upper bound rather than as the operating estimate.

The per-metric pattern is interpretable (Table 7 and Table 9). Trunk lean and head tilt are recovered at near-expert agreement ($F_{1}=0.92$ and 0.94 against consensus). Inter-limb asymmetry rises from $F_{1}=0.66$ and 0.60 against the two individual raters to 0.73 against the consensus, indicating that its lower per-rater score is driven by the repetitions on which the clinicians themselves disagree rather than by a detector defect. Incomplete elbow extension, at $F_{1}=0.72$ (recall 0.69 at precision 0.74), recovers to a usable range once the arm-elevation and elbow angles are measured in the corrected, aspect-ratio-independent coordinates (Section 3.3), though its operating point remains cohort-sensitive (Section 4.6). Shoulder elevation stays the weakest sign even on the unambiguous consensus subset ($F_{1}=0.57$, precision 0.53). Its false-positive rate against the clinician labels falls from 0.33 to 0.15 (Table 10), and its flag rate on repetitions *instructed* to be compensation-free falls from ∼41% to ∼14% (Table 7). That gain is attributable to the recalibration rather than to the change of coordinates: this metric is a ratio of a vertical displacement to the shoulder width, so the isotropic correction rescales it by a near-constant factor and leaves its ranking, and hence its discrimination, essentially unchanged—at the operating point geometrically equivalent to the submitted one, the corrected data reproduce the previous error rates. What the recalibration bought is precision at the cost of recall, at almost unchanged $F_{1}$; a residual confound remains—a direct consequence of frontal-monocular projection, in which shoulder rise is geometrically entangled with the abduction motion itself. This is the detector’s one genuine limitation among the five, separable here from the inherent ambiguity of inter-limb asymmetry. Until a stereo or inertial channel can resolve the projection confound, the shoulder-elevation flag should be regarded as research-grade rather than a deployable clinical criterion, whereas the trunk-lean and head-tilt flags already operate at near-expert agreement. Restricted to these two near-expert flags, the macro-$F_{1}$ is 0.93; adding the two moderate flags (elbow extension and inter-limb asymmetry) gives 0.83 across four, with shoulder elevation the single research-grade exception, so the five-metric macro-average of 0.78 blends signs of differing maturity and should not be read as a single deployable-performance figure.

**Table 7:** System flag rate per *instructed* condition: each entry is the fraction of that row’s *n* repetitions in which the column’s flag fired. Rows are the instructed compensation, columns the detector’s five flags; the diagonal entry of each row is that condition’s target metric (detection). Because participants were not required to isolate the instructed compensation and the clinicians labeled every sign they observed, off-diagonal mass mixes two causes: genuine co-occurring compensations and detector confusions. This table alone cannot separate them—the label-based false-positive rates in Table 10 can, and they identify shoulder elevation (0.15) as the one flag with a substantial residual confusion, which is much reduced after the isotropic coordinate correction (Section 3.3) but not eliminated.

Condition	Elbow	Asym.	Shoulder	Trunk	Head
Correct (*n* = 177)	0.01	0.03	0.14	0.00	0.00
Elbow (*n* = 89)	0.65	0.39	0.20	0.00	0.03
Asymmetry (*n* = 90)	0.08	0.86	0.28	0.04	0.02
Shoulder (*n* = 89)	0.08	0.00	0.73	0.00	0.00
Trunk (*n* = 89)	0.02	0.18	0.00	1.00	0.12
Head (*n* = 87)	0.00	0.23	0.17	0.10	0.97

**Table 8:** *Primary analysis.* Per-class $F_{1}$ against *each* clinician over all 621 repetitions with 95% confidence intervals (participant-stratified bootstrap, 10,000 resamples). This whole-set, per-rater evaluation—not the consensus subset—is the operating estimate; the two raters give consistent per-class orderings.

Compensation	$F_{1}$ vs. Rater 1	$F_{1}$ vs. Rater 2
Incomplete elbow extension	0.72[0.54,0.86]	0.73[0.56,0.86]
Inter-limb asymmetry	0.66[0.57,0.75]	0.60[0.53,0.67]
Shoulder elevation	0.55[0.43,0.68]	0.50[0.39,0.63]
Trunk lean	0.92[0.85,0.98]	0.92[0.84,0.98]
Head tilt	0.90[0.86,0.94]	0.87[0.83,0.91]
**Macro**	0.75[0.69,0.80]	0.72[0.66,0.78]

**Table 9:** Per-class detection performance against the two-clinician consensus reference (the 477 repetitions of full inter-rater agreement; 18 participants). *Overall* is the macro-average across the five metrics.

Compensation	Precision	Recall	$F_{1}$
Incomplete elbow extension	0.74	0.69	0.72
Inter-limb asymmetry	0.67	0.81	0.73
Shoulder elevation	0.53	0.63	0.57
Trunk lean	0.84	1.00	0.92
Head tilt	0.91	0.97	0.94
**Overall**	0.74	0.82	0.78

**Table 10:** Full per-class metric panel against the two-clinician consensus reference (477 repetitions), each with a 95% confidence interval (participant-stratified bootstrap, 10,000 resamples). Recall equals sensitivity for a binary flag; specificity and the FP-rate are computed over the repetitions the clinicians labeled negative *for that particular sign*, so their denominators differ from class to class (FP-rate =1−specificity).

Compensation	Precision	Recall (Sens.)	Specificity	FP-Rate	$F_{1}$
Incomplete elbow extension	0.74[0.54,1.00]	0.69[0.51,0.87]	0.96[0.92,1.00]	0.04[0.01,0.08]	0.72[0.55,0.86]
Inter-limb asymmetry	0.67[0.57,0.79]	0.81[0.70,0.90]	0.90[0.86,0.94]	0.10[0.06,0.14]	0.73[0.64,0.82]
Shoulder elevation	0.53[0.39,0.74]	0.63[0.47,0.79]	0.85[0.74,0.94]	0.15[0.06,0.26]	0.57[0.45,0.71]
Trunk lean	0.84[0.68,1.00]	1.00[1.00,1.00]	0.98[0.96,1.00]	0.02[0.00,0.04]	0.92[0.81,1.00]
Head tilt	0.91[0.84,1.00]	0.97[0.93,1.00]	0.985[0.97,1.00]	0.015[0.00,0.03]	0.94[0.90,0.98]

### 4.5. Real-Time Performance

On-device throughput was measured during live exercise on six consumer laptops spanning several CPU generations and vendors (Table 11). These runs were logged as feasibility checks rather than as a controlled benchmark: the application build and the requested camera settings were common to all six machines, but the operating-system and browser versions, the delivered camera resolution, and the execution backend actually used were not recorded per device, so the numbers below characterize the achievable range on ordinary hardware and should not be used to compare devices or browsers against one another. The full “frame → pose → metrics → feedback” loop sustains 22–30 FPS across the panel—from 22 FPS on an older Ryzen 5 ultrabook to 30 FPS on the Apple M1—at a moderate CPU load of 9–20% and without any reliance on a discrete GPU with integrated graphics contributing at most ∼12% where present. These rates clear the ≥25 FPS real-time target on the modern configurations and remain in the responsive range on mid-tier hardware, which establishes that interactive, markerless feedback is feasible on everyday machines rather than dedicated equipment. The sustained frame rate corresponds to a per-frame budget of roughly 33–45 ms, which is consistent with the backbone inference characterized in Section 3.2. Two latencies must be distinguished. The *computational* latency—from the frame in which the violation condition is satisfied to the on-screen notification—is below 120 ms, and it is what determines whether the display keeps up. The *total detection delay*—from the physical onset of a compensation to the displayed feedback—is necessarily larger, because a violation must persist for the 250 ms accumulation window (Section 3.5) before a repetition is flagged; the total delay is therefore at least this window plus the computational and display latency—on the order of 350–400 ms. The notification therefore arrives within a typical repetition rather than after it: across the 621 detection repetitions, the lift/hold/lower cycle had a median duration of 1.4 s (interquartile range 1.2–1.6 s), so a 350–400 ms delay falls within roughly the first third of the movement. The <120 ms figure is an upper bound observed during operation; a formal latency protocol (fixed instrumentation points, a large number of trials, and mean/median/worst-case reporting on named hardware) is left to a dedicated deployment study.

### 4.6. Threshold Sensitivity Analysis

**Figure 4:**
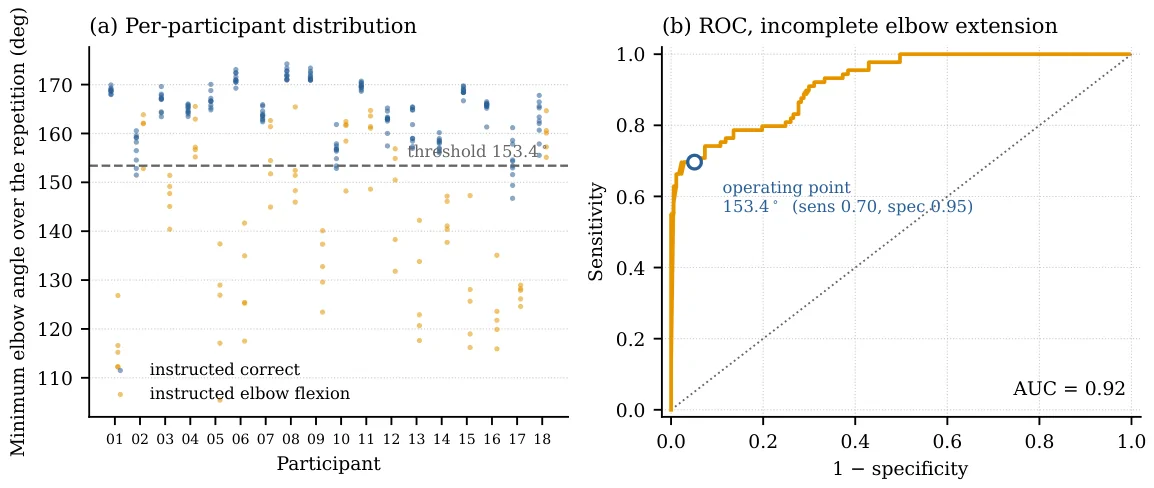
Behavior of the incomplete-elbow-extension criterion on the detection cohort. (**a**) Minimum elbow angle reached in each repetition, per participant, for repetitions instructed to be compensation-free (blue) and instructed to contain elbow flexion (orange); the dashed line is the calibrated threshold. (**b**) ROC curve for the same quantity with the calibrated operating point marked. Both panels use the instructed condition as the reference and the raw per-repetition angle without the 250 ms persistence rule applied by the full detector.

Because the detector uses empirically calibrated preliminary thresholds rather than learned decision boundaries, its robustness to the exact threshold values was examined on the consensus reference. Each of the five thresholds in Table 3 was varied independently by ±20% around its nominal value, with the other four held fixed, and the per-class $F_{1}$ was recomputed from the raw keypoints (Table 12). Three of the five metrics sit on a stable plateau—inter-limb asymmetry, shoulder elevation, and head tilt each stay within ±0.05 of their nominal $F_{1}$ across the full ±20% sweep. Trunk lean varies more widely (spanning 0.75 to 0.98, a 0.23 range) but never drops below a usable 0.75, degrading gracefully rather than sitting flat. Their reported performance is therefore not an artifact of finely tuned constants. The incomplete-elbow-extension threshold is the exception: tightening it by 20% ($153^{\circ }\;\to \;122^{\circ }$) drops elbow $F_{1}$ from 0.72 to 0.23, because the simulated elbow-flexion repetitions cluster just below the nominal cutoff, so a stricter angle no longer reaches them. Because the simulated repetitions cluster near the cutoff, the nominal elbow $F_{1}$ is itself specific to this healthy-volunteer distribution and should not be read as an estimate of real post-stroke incomplete-extension detection until recalibrated on a patient cohort. Although the elbow criterion recovered to a usable range after the coordinate correction (Section 4.4), its operating point remains the one constant that genuinely requires care and would most benefit from per-cohort calibration, whereas the displacement-based metrics tolerate moderate threshold variation. The underlying separation is nonetheless clear on this cohort (Figure 4). Taking the instructed condition as the reference, the minimum elbow angle has a median of $142^{\circ }$ on elbow-flexion repetitions versus $166^{\circ }$ on those instructed to be compensation-free, with an area under the ROC curve of 0.92; at the calibrated operating point of $153.4^{\circ }$, this gives a sensitivity of 0.70 at a specificity of 0.95. These characterize the raw angle threshold applied per repetition, without the 250 ms persistence window that the full detector additionally requires, which is why they differ from the firing rates of Table 7. Panel (a) also shows why the threshold is fragile: the two conditions overlap substantially, and several participants produced elbow-flexion repetitions well above the cutoff. The difficulty is therefore one of *threshold placement* on a narrow healthy-volunteer distribution rather than of discriminative power.

Finally, the sweep varies one threshold at a time; it therefore does not probe interactions between thresholds, and joint variation could in principle behave differently from the marginal sweeps reported here.

## 5. Discussion

This paper set out to determine whether a fully on-device, markerless pipeline can recover upper-limb rehabilitation kinematics with measurement accuracy adequate for movement monitoring while running on everyday hardware—a technical proof-of-concept rather than a demonstration of clinical effectiveness, which would require a patient study. The validation supports a qualified affirmative on three counts, whose numbers are reported in Section 4 and are not repeated here.

First, the image-plane abduction angle conforms to every pre-specified metrological bound against the inertial reference over the range the recordings actually cover. Two qualifications bound that result. The summaries describe average error, whereas the limits of agreement—which bound a single measurement—are correspondingly wider, and a total-agreement limit pooling within- and between-subject variance was never computed (Section 3.6). And the result validates the arm-elevation angle and *only* that angle: the four non-angular flags are not measured by the IMU at all and rest entirely on clinician vision, so the strong agreement must not be read as evidence for the detector as a whole.

Second, the complete “frame → pose → metrics → feedback” loop runs at interactive rates on six consumer laptops without relying on a discrete GPU, which establishes feasibility on commodity machines. Third, the pipeline executes client-side, so the raw patient video never leaves the device—though, as discussed below, the derived keypoints that may be exported are not themselves privacy-neutral.

For the compensation detector, the contribution is the design: five normalized metrics evaluated against empirically calibrated preliminary thresholds and pooled into an unvalidated summary score *Q*. We validate the five constituent flags—not *Q*—against two clinicians who were independent of the acquisition sessions and blind to the instructed condition, so the reference labels are not anchored to the experimental design. The per-metric pattern, rather than the average, is what matters: trunk lean and head tilt reach near-expert agreement; inter-limb asymmetry is bounded by genuine ambiguity between the raters themselves; incomplete elbow extension recovered to a usable range once the coordinates were made aspect-ratio-independent; and shoulder elevation remains the detector’s own limitation under frontal-monocular projection even after that correction substantially reduced its false-positive rate. Because the compensations were simulated by healthy volunteers, we frame the thresholds as preliminary operating points rather than a finished clinical accuracy result.

To place the angular agreement in context—rather than to rank it against studies that are not directly comparable—we list a few reference points from the literature, each obtained on a different joint, task, or reference modality. The closest methodological match is monocular on-device upper-limb angle estimation validated against a clinical goniometer, which reports a shoulder RMSE of $3.21^{\circ }$ and an elbow RMSE of $3.65^{\circ }$ [24]; our RMSE of $2.61^{\circ }$ is of a similar order, though the two are not a like-for-like comparison, being bounded by their small healthy sample and static range-of-motion endpoints versus our continuous inertial trajectory. The broader markerless literature sets a comparable bar: two-dimensional video against optical motion capture yields per-joint MAEs of roughly 4–$7^{\circ }$ for lower-limb gait ($4.0^{\circ }$ hip, $5.6^{\circ }$ knee, $7.4^{\circ }$ ankle) [14], multi-camera markerless capture reports rotational MAEs near $4.5^{\circ }$ against marker-based reference [15], and monocular joint-angle agreement with a goniometer is commonly cited below $2.5^{\circ }$ [16]. Our $2.18^{\circ }$ MAE for seated shoulder abduction falls in the same broad range as these values, which we present only as contextual anchors: they differ in joint, task, and reference modality, so the comparison indicates plausibility rather than any ordering of methods. The result is also notable for the joint it targets: prior depth-sensor work found shoulder range of motion among the least reliable upper-limb measurements (poor-to-moderate reliability for elbow and shoulder ROM in seated reaching) [17], which is precisely the regime where our image-plane estimate performs well. The agreement also compares favorably with routine clinical practice, where manual goniometry carries inter-rater variation of several degrees, especially at the upper limb [8,9]. We deliberately report image-plane angles rather than monocular three-dimensional positions, because single-camera positional reconstruction remains noisy—median errors near 56 mm that stereo fusion only halves to about 30 mm [21]—so an angular formulation in the movement plane is the more reliable target. What distinguishes the present system from these comparators is not any single number but the combination they lack: fully on-device execution, a published set of fixed-threshold compensation metrics, and concurrent validation against both an inertial equivalence reference and blinded clinician ratings. The thresholds were, moreover, fixed on a calibration cohort disjoint from the participants used for detection, so the reported figures are across-subject rather than within-subject. That distinction is not academic: a webcam-based deep compensation classifier retained an accuracy of 0.92–0.95 within participants but only about 0.70 across them [49]. Prior efforts deliver accurate kinematics without reproducible compensation detection [19,20,23], or they offer compensation detection tied to specialized hardware and per-setup learned thresholds [43,45,46,47].

Running entirely client-side matters for telerehabilitation rather than being incidental. Because frame capture, pose estimation, and metric computation all execute locally, raw video is never transmitted or stored remotely in the deployed configuration, which removes a category of privacy exposure that is acute for identifiable medical recordings [38]. We are careful not to overstate this. The present dataset was collected with a research instrument that deliberately *did* record and store video, because blinded clinician annotation requires it (Section 3.7); the on-device property describes the patient-facing pipeline—not the study apparatus. Even in the deployed configuration, two qualifications remain: the model weights and runtime are fetched from a public CDN before inference, and the derived keypoint stream—exportable, unencrypted, and health-related—is not privacy-neutral merely because the video stayed local. Local execution also eliminates the hard connectivity dependency and round-trip latency of a server-side estimator, allowing corrective feedback within the same repetition, and it lowers the barrier to entry: the system needs only a browser, a webcam, and integrated graphics, in contrast to the controlled environment and recurring cost of marker-based optical systems or the calibration burden of multi-sensor inertial setups.

Several limitations bound these findings.

- **Monocular two-dimensional projection.** The angle is computed in a single image plane, so agreement depends on a frontal camera placement in which the movement unfolds parallel to the sensor; out-of-plane motion and camera misalignment would degrade it, and the method recovers neither depth nor kinetics (forces and moments). Validation is confined to a *single* exercise—seated frontal-plane shoulder abduction—so the reported segmentation, thresholds, and metric performance should not be generalized to upper-limb rehabilitation in general. Functional reaching, out-of-plane movements, and other directions each require separate verification; these are a priority for future work.
- **Validation scope.** The inertial equivalence study used ten healthy participants performing five repetitions each (fifty cycles) on a single standardized task; while adequate for a feasibility estimate of agreement, this sample does not capture the full variability of clinical populations or movements.
- **Simulated compensations.** The detection framework was evaluated on compensations performed deliberately by healthy volunteers, which are cleaner and more exaggerated than the subtler, more variable pathological compensations seen after stroke; performance on genuine clinical presentations may therefore differ. Because the strongest metrics (trunk lean and head tilt) are large-amplitude gestures most easily exaggerated on demand, their near-expert agreement is also the most likely to regress on subtler genuine compensations. The protocol mitigated this only partly: the compensations were demonstrated by a clinician rather than defined by an instrumented amplitude target, and participants were free to produce co-occurring compensations rather than isolated ones (Section 4.4). Genuine post-stroke movement additionally involves a restricted range of motion, spasticity, abnormal synergies, and variable speed, none of which a healthy volunteer reproduces on request.
- **Coupled compensations.** Several patterns co-occur physiologically: bending the elbow or leaning the trunk also displaces the shoulders and arms, so shoulder elevation and inter-limb asymmetry are not fully independent of the other criteria. We mitigate the most obvious confounds with explicit suppression rules (Section 3.5) but do not hard-decouple the patterns; instead, the residual coupling is assessed through agreement with clinician annotation. Consistent with this, shoulder elevation remains the weakest criterion in the validation (Section 4.4), still firing on a minority of compensation-free repetitions, while incomplete elbow extension—the other metric affected by coupling—recovered to a usable range once the coordinates were made aspect-ratio-independent, though it stays the most threshold-sensitive of the five.
- **Preliminary, population-general thresholds.** The five thresholds are calibrated on a pilot reference set and frozen before validation on explicit geometric grounds rather than learned end-to-end. They remain population-general and preliminary: the reported operating points await confirmation on a separate clinical cohort of real patients, and per-patient calibration may further help individuals whose resting posture or range deviates from the reference assumptions.
- **Image-quality sensitivity.** Like all monocular pose estimation, the pipeline depends on keypoint quality, so poor lighting, low-resolution cameras, and partial occlusion can perturb the underlying landmarks and, in turn, the derived metrics.
- **Population and equipment coverage.** BlazePose, like most pose estimators, is trained predominantly on able-bodied adults, so its landmark accuracy may degrade for underrepresented body types, ages, or skin tones as well as for patients using wheelchairs, slings, orthoses, or other assistive devices. We did not test participants with assistive equipment, limb deformities, or severe movement restriction, and the system’s behavior in those settings is unknown. The sample is also young (18–32 years) and predominantly male (≈75%), so age- and sex-related variation in movement strategy is barely sampled.
- **The aggregate score *Q* is unvalidated.** *Q* pools the five per-repetition flags with equal weights, so its only validated content is those flags; the weighting itself was not derived from data. It has not been compared against clinical scales, impairment severity, or functional outcome, and nothing in this study establishes that a change in *Q* corresponds to a change a clinician would regard as meaningful. It is reported as an exploratory summary and should not be read as a clinical measure.
- **The segmentation itself is unvalidated.** Every reported detection figure is conditioned on the repetitions the finite-state machine produced: its boundaries define the interval over which each criterion is checked. We never validated that segmentation against an independent reference—neither manual marking of repetition boundaries nor a sensor-derived one—so repetition-count accuracy and phase-boundary error are unknown, and a systematic bias in the phase boundaries would propagate into every metric without appearing as a detector error. Establishing this requires an independently annotated reference that the present dataset does not contain.
- **Bilateral field of view is required.** Inter-limb asymmetry and shoulder elevation are defined on both shoulders, so both arms must remain in frame throughout (Section 3.7). A unilateral setup, an occluded contralateral arm, or a patient who can only move one side falls outside the current scope—a real restriction for hemiparetic use, which is exactly the target population.
- **Device independence is shown analytically, not measured.** The aspect-ratio invariance of Table A1 is a re-projection of one recorded dataset: it establishes that the corrected metric definitions cannot depend on the frame aspect ratio, which is a property of the formulation rather than an empirical result. Every recording in this paper came from a single camera configuration (1280×720 at 30 FPS), so the effects that actually accompany a change of device—optics, sensor noise, rolling shutter, lens distortion, autoexposure behavior—remain untested. A multi-camera comparison is required before device independence is claimed empirically.
- **The angular validation is offline, not causal.** The inertial comparison applied zero-phase filtering and post hoc cross-correlation alignment, neither of which exists in the deployed system, whose smoothing is a causal exponential moving average with a group delay of about 62 ms. The reported agreement therefore characterizes the geometric estimator with timing effects removed and should not be read as the accuracy a user experiences instant by instant; the discrepancy falls on the transient lift and lower phases rather than on the hold plateau that fixes the amplitude. Quantifying the causal pipeline against an instrumental reference—which the retained data no longer permit—is necessary before real-time angular readings are used for anything beyond feedback.
- **The angular validation covers only part of the range, and the error is amplitude-dependent.** The participants did not raise the arm to the horizontal: the recorded trajectories plateau near $61^{\circ }$, so agreement with the inertial reference is established over roughly 0–$62^{\circ }$ rather than the full abduction range. This matters more than a simple coverage gap because the difference is not constant—it grows at about 4% of the measured angle (Section 4.3). Extrapolating the reported accuracy to larger elevations is therefore not supported: the same trend projected to $90^{\circ }$ would imply a systematic component of roughly $4^{\circ }$, which is beyond the tolerance the method meets within the measured interval. Validation across the full clinical range, and a check of whether a single scale correction removes the trend, are needed before the angle is used at larger amplitudes.
- **Deployment measurements are feasibility checks, not a benchmark.** The throughput figures were collected on six laptops with a common application build, but the operating-system and browser versions, the delivered camera resolution, and the execution backend were not logged per device, and end-to-end latency was not measured under a fixed protocol (Section 4.5). The reported rates therefore establish that interactive operation is attainable on commodity hardware without supporting any comparison between devices, browsers, or backends; a properly instrumented deployment study is needed for that.
- **Anthropometric robustness is by design, not demonstrated.** The metrics are constructed to limit body-size dependence—they are angles, or displacements referenced to the participant’s own resting shoulder width—and the participants spanned roughly 165–190 cm and 60–120 kg. However, anthropometry was not systematically measured (Section 4.1), so we cannot regress metric values or detection performance on stature, mass, or limb length, and the subgroup analysis that would substantiate body-size invariance is not possible on this dataset. Handedness was likewise not recorded, which matters specifically for the inter-limb asymmetry metric: a systematic difference between the dominant and non-dominant arm would appear as asymmetry, and we cannot separate that contribution from the simulated compensation. Recording basic anthropometry and handedness is a straightforward addition to the next study.

These limitations also chart the path forward. The shoulder-elevation confound in particular admits several remedies short of new hardware: referencing one shoulder’s rise to the contralateral shoulder rather than to the mean shoulder line, gating the metric to the specific movement phase in which a true shrug (but not the projection artifact) appears, or using a participant-specific baseline range instead of a single fixed threshold; adding a depth, stereo, or inertial channel would resolve it more directly. More broadly, fusing the image-plane estimate with a lightweight inertial channel, or extending to calibrated three-dimensional pose, would relax the frontal-plane constraint and broaden the set of measurable movements while preserving the on-device footprint. Adaptive, per-patient thresholds—learned from a short calibration sequence—could personalize the detector without sacrificing the reproducibility of the fixed-threshold baseline. The most important next step is a larger, longer clinical study on post-stroke and other patient cohorts to confirm stability across populations and to relate the measured kinematics to functional recovery; the blinded two-clinician annotation study reported here is a first step, but it was conducted on healthy volunteers simulating the compensations. Such a study also inherits the limits of its reference: therapists rating compensation from video agree only moderately, and markedly less on global movement quality than on individual task phases, so clinician labels bound the agreement any detector can demonstrate against them and are themselves best paired with instrumented measurement [10]. Standards-based integration with electronic health records (e.g., HL7 FHIR) is a plausible longer-term direction but was not evaluated here.

## 6. Conclusions

This paper presented a markerless, fully on-device system that recovers upper-limb rehabilitation kinematics from a single frontal RGB camera and turns them into interpretable feedback expressed in clinically recognizable terms (whose effectiveness in care remains to be established) without any server-side computation. The pipeline couples a BlazePose backbone with a compact set of five normalized compensation metrics (designed to limit body-size dependence) and an aggregate quality score *Q*—an unvalidated, equal-weighted convenience summary—all evaluated against empirically calibrated preliminary thresholds. Checked against a synchronized inertial reference on a standardized seated shoulder abduction task, the image-plane angle met every pre-specified metrological bound over the 0–$62^{\circ }$ range the recordings cover (median MAE $2.18^{\circ }$, Lin’s concordance correlation coefficient 0.956), although the difference grows in proportion to the angle, so the accuracy should not be extrapolated to larger elevations. The complete pipeline sustained 22–30 FPS on six consumer laptops without relying on a discrete GPU. The compensation detector is provided as a reproducible fixed-threshold framework whose agreement with clinician judgment was assessed in a blinded two-clinician study, reaching a macro-averaged $F_{1}$ of 0.75 and 0.72 against the individual raters (0.78 against their consensus subset, an upper bound)—near-expert for trunk lean and head tilt, moderate for incomplete elbow extension (which recovered once angles were measured in aspect-ratio-independent coordinates) and inter-limb asymmetry, and weakest for shoulder elevation; all of this has been established so far only on healthy volunteers simulating the compensations. By keeping the raw video on the device, exporting only derived data (keypoints and metrics), and running on commodity hardware, the system targets the cost, privacy, and infrastructure barriers that have limited objective motor assessment outside the clinic, offering a proof-of-concept foundation for accessible home telerehabilitation rather than a finished clinical tool. Future work will extend the approach with inertial or three-dimensional fusion (expected to mitigate the shoulder-elevation confound), per-patient adaptive thresholds, and a study on genuine post-stroke compensations linking the measured kinematics to functional recovery.

## Figures and Tables

**Table 1:** Positioning against the closest prior compensation-analysis systems, summarizing this section. Only the present system combines fully in-browser on-device execution, fixed thresholds that are not re-fitted per setup, and concurrent IMU-plus-clinician validation. n/r: not reported in the cited work. The last column gives each work’s own headline detection figure as published, and these figures are *not* comparable with one another, because each is measured against a different reference: ours against the independent judgement of two clinicians on every repetition, ref. [43] against the instructed task condition rather than against an observer, and ref. [45] against compensation labels whose provenance the report does not state. Ref. [48] reports keypoint-localization accuracy (F1 0.85–0.92 against the authors’ own annotations) but no compensation-detection result, and refs. [46,47] report none for the systems as cited. The reference standard, not the number, is what these rows are best read along.

System	Modality	On-Device	Detection	Thresholds	Validation	Reported
This work	Monocular RGB	In-browser	Five metrics	Fixed, dev.-indep.	IMU + clinicians	F1 0.75/0.72
[48]	Monocular RGB	n/r	Asym./comp.	Hand-set bins	n/r	n/r
[46]	Depth (Kinect)	Desktop	Yes	Fixed	Feedback study	n/r
[47]	Depth (Kinect)	Desktop	Seq. models	Learned	Public dataset	n/r
[45]	IMU (wearable)	Wearable	Scores	Learned	Post-stroke	acc. 95.7%
[43]	Pressure mat	Instrumented	Yes	Learned	Stroke surv.	F1 0.993

**Table 2:** Accuracy and inference characteristics of candidate HPE models. The accuracy columns (mAP, PCK@0.2) are values *reported in the literature* on COCO by the respective sources and are collected here only as contextual reference points rather than as a controlled head-to-head benchmark—they were obtained on different datasets and hardware, so small differences should not be read as a definitive ranking. The inference rate (Inf. FPS) is single-model throughput measured for the backbone in isolation; the latency column is the corresponding per-frame budget derived from that rate (1000/FPS) rather than an independently instrumented frame-to-coordinates measurement. Both are *distinct* from the end-to-end pipeline rate on deployment hardware (22–30 FPS, Section 4.5), which additionally includes capture, normalization, metric computation, and rendering. The heaviest variant, BlazePose Heavy, is omitted here, as it requires a discrete GPU to stay within the real-time budget.

Model	mAP	PCK@0.2	Size (MB)	Latency (ms)	Inf. FPS
OpenPose [30]	65.3	76.3	–	102	9.8
MoveNet Thunder	74.1	–	12.6	13	77
MoveNet Lightning	59.2	–	2.9	10	104
PoseNet (MobileNetV1)	45.6	–	13.3	13	80
BlazePose Full [33]	65.9	95.5	10.6	12	81
BlazePose Lite	50.8	90.2	3.5	11	92

**Table 3:** Compensation metrics: clinical meaning, geometric quantity, and *preliminary* threshold. φ is the trunk-lean angle; the asymmetry and head-tilt flags are additionally suppressed unless the trunk is upright (footnote). Thresholds were calibrated on the nine-participant calibration cohort and frozen before validation (see “Threshold Calibration”); they are preliminary, pending a fuller pilot.

Compensation	Clinical Interpretation	Quantity	Flag (Prelim.)
Incomplete elbow extension	Substituting elbow flexion for shoulder abduction	$\theta_{\mathrm{elb}}^{\min}$ (Equation (7))	<$153.4^{\circ }$
Inter-limb asymmetry	Unequal elevation of the two arms	ΔΘ (Equation (5))	≥$13.2^{\circ }$
Shoulder elevation	Compensatory shrug of the shoulder girdle	*s* (Equation (8))	≥ 0.174
Trunk lean	Leaning the torso to raise the arm	φ (Equation (9))	≥0.090 rad
Head tilt	Lateral neck/head tilt during effort	η (ear-line)	≥$6.4^{\circ }$

Violation window 250 ms; trunk-suppression threshold 0.072 rad.

**Table 4:** The three disjoint participant samples. No individual appears in more than one sample.

Sample	Purpose	*N*	Data
Angular validation	Accuracy vs. inertial reference	10	50 cycles
Threshold calibration	Setting and freezing thresholds	9	292 repetitions
Detection evaluation	Agreement with clinician labels	18	621 repetitions
**Total**		**37**	

**Table 5:** Group agreement metrics summarized *within* subjects, then across the 10 subjects (median [Q1–Q3] of the per-subject values, each computed over that subject’s five repetitions). Here, “LoA width” is the full span between the upper and lower 95% limits of agreement of a single subject; the value below is the median of those per-subject spans. These per-subject limits are a different quantity from the limits shown in Figure 3 ($-0.87^{\circ }$ to $+3.68^{\circ }$, a span of $4.55^{\circ }$): the figure aggregates the cycle *averaged across participants*, so between-subject scatter is removed and its limits are correspondingly narrower. Neither is a formal repeated-measures estimate (Section 3.6).

Metric	Median [Q1–Q3]
MAE (°)	2.18 [1.62–2.83]
RMSE (°)	2.61 [2.14–3.19]
Bias (°)	0.92 [0.38–1.47]
LoA width, per-subject (°)	7.35 [6.21–8.58]
Lin’s CCC ^†^	0.956 [0.942–0.967]

^†^ The CCC bracket was reported as a 95% confidence interval in the original angular-validation analysis, unlike the other rows, whose brackets are across-subject interquartile ranges; the construction of that interval is not documented and the values are reproduced as obtained.

**Table 6:** Interval-based assessment against the pre-specified metrological bounds. Equivalence is read as containment of the interval estimate within the bound. Brackets are interquartile ranges across the ten per-subject values, not confidence intervals; the comparison is therefore descriptive and is not a formal equivalence test.

Quantity	Median [Q1–Q3]	Bound
Signed bias (°)	0.92 [0.38,1.47]	within ±2
MAE (°)	2.18 [1.62,2.83]	below 5
Lin’s CCC	0.956 [0.942,0.967]	—

**Table 11:** Client-side performance on consumer devices during exercise. The application build was identical on every machine—MediaPipe Tasks Vision 0.10.14 with the pose_landmarker_full (float16) model, the GPU delegate requested over the WebAssembly runtime, single-pose VIDEO mode—and the camera was requested at 1280×720 and 30 FPS. What was *not* recorded per device is the operating-system and browser version, the resolution the webcam actually delivered, and whether the requested GPU delegate was in fact granted rather than falling back to CPU execution; a dash in the GPU column likewise means that utilization was not recorded on that machine. This panel should therefore be read as a set of feasibility checks that the pipeline sustains interactive rates on ordinary hardware—not as a controlled comparison between devices or browsers.

Device	CPU	CPU%	GPU%	FPS
HP Omen 15-ce072ur	i7-7700HQ	∼20	∼12	27
Honor MagicBook X 15	Ryzen 5 2500U	∼14	–	22
Honor MateBook D16	i5-12500H	∼12	–	28
Acer Aspire A5	i5-9700	∼15	∼10	25
MacBook Pro 13” (M1)	Apple M1	∼9	–	30
Lenovo ThinkPad T14s	Ryzen 7 PRO 4750U	∼13	∼7	27

**Table 12:** Per-class $F_{1}$ against the consensus reference as each threshold is varied by ±20%—one at a time with the other four held at their nominal value. Only incomplete elbow extension is threshold-sensitive.

Metric (Nominal Threshold)	−20%	Nominal	+20%
Incomplete elbow extension ($153.4^{\circ }$)	0.23	0.72	0.23 ^†^
Inter-limb asymmetry ($13.2^{\circ }$)	0.69	0.73	0.74
Shoulder elevation (0.174)	0.55	0.57	0.54
Trunk lean (0.090 rad)	0.75	0.92	0.98
Head tilt ($6.4^{\circ }$)	0.90	0.94	0.95

^†^ +20% raises the elbow cutoff to $184^{\circ }$, above the anatomical maximum of $180^{\circ }$, so every repetition is flagged; the meaningful sensitivity is the −20% direction.

## Data Availability

The repetition-level data and the analysis code are publicly archived at https://doi.org/10.5281/zenodo.21747009 (version 1.1.0, CC BY 4.0): per-repetition metric summaries for all 913 repetitions, both clinicians’ independent annotations, the calibrated thresholds, scripts that recompute every value reported here and check it against the manuscript, and the study instrument itself—minus its live configuration, which held access tokens and the annotators’ names and is replaced by a placeholder. Because a flag is raised only once a violation persists for the accumulation window, these summaries are a sufficient statistic for that rule, so the detector’s output is recoverable at any threshold. The de-identified per-frame keypoints are available on request from the corresponding author: the consent obtained covers publication of anonymized results, not of continuous per-person kinematic records. The video recordings are not publicly available at all—identifiable video of participants held under pseudonymous codes on a restricted laboratory server is outside the scope of the consent given. The raw inertial recordings of the earlier, separate angular-validation study are no longer retained.

## Abbreviations

The following abbreviations are used in this manuscript:

HPE	Human Pose Estimation
ROM	Range of Motion
IMU	Inertial Measurement Unit
MAE	Mean Absolute Error
RMSE	Root Mean Square Error
LoA	Limits of Agreement
CCC	Concordance Correlation Coefficient (Lin)
EMA	Exponential Moving Average
FPS	Frames Per Second

## Appendix A. Supporting Tables

This appendix collects reference material referred to from the main text: the aspect-ratio invariance check (Section 3.3), the segmentation thresholds (Section 3.4), the phase-stratified agreement of the angular validation (Section 4.3), and two supporting breakdowns of the detection study (Section 4.4).

**Table A1:** Maximum absolute deviation of each metric from its native-resolution value when the same pose is normalized at different aspect ratios before (per-axis x/W,y/H) and after (isotropic x/H,y/H) the correction. Angles in degrees; the shoulder metric is the shoulder-width-normalized rise.

	Per-Axis (Uncorrected)					Isotropic (Corrected)				
Aspect Ratio	Arm Elev.	Elbow	Trunk	Head	Shoulder	Arm Elev.	Elbow	Trunk	Head	Shoulder
4:3	$8.2^{\circ }$	$9.8^{\circ }$	$3.0^{\circ }$	$8.1^{\circ }$	0.75	$<0.001^{\circ }$	$<0.01^{\circ }$	$<0.001^{\circ }$	$<0.001^{\circ }$	<0.001
1:1	$0^{\circ }$	$0^{\circ }$	$0^{\circ }$	$0^{\circ }$	0	$0^{\circ }$	$0^{\circ }$	$0^{\circ }$	$0^{\circ }$	0
21:9	$24.0^{\circ }$	$36.0^{\circ }$	$7.0^{\circ }$	$24.0^{\circ }$	3.08	$<0.001^{\circ }$	$<0.01^{\circ }$	$<0.01^{\circ }$	$<0.01^{\circ }$	<0.001

**Table A2:** Finite-state-machine thresholds for phase segmentation. All are fixed geometric values on the arm-elevation angle Θ and its speed $\dot{\Theta }$, referenced to the calibrated functional zero.

Transition	Threshold	Role
rest → lift	$\Theta \geq 25^{\circ }$	repetition start $t_{s}$
lift → hold	$\Theta \geq 55^{\circ }$, $|\dot{\Theta }|<\omega_{\mathrm{th}}$	top of the movement
hold → lower	$\dot{\Theta }<-\omega_{\mathrm{th}}$	descent begins
lower → rest	$\Theta <18^{\circ }$	repetition end $t_{e}$
speed threshold	$\omega_{\mathrm{th}}=20^{\circ }/s$	lift/hold/lower gating

These four constants define phase boundaries rather than detection criteria, and unlike the five metric thresholds, they were *not* re-derived after the coordinate correction: no criterion exists against which to re-select them, since the segmentation was never validated against an independent reference (Section 5). Because the correction shifts the scale of Θ, the boundaries correspond to slightly different elevations than in the submitted version; that shift is absorbed in the full recomputation, from which every reported detection figure is derived. The $25^{\circ }$/$18^{\circ }$ hysteresis rejects momentary dips at the bottom of the movement.

**Table A3:** Phase-stratified agreement (median [Q1–Q3] across subjects).

Phase	MAE (°)	RMSE (°)	Bias (°)
Lift	2.45 [1.92–3.08]	2.98 [2.46–3.56]	0.98 [0.44–1.58]
Hold	1.64 [1.22–2.06]	2.02 [1.66–2.43]	0.71 [0.28–1.09]
Lower	2.72 [2.18–3.31]	3.21 [2.72–3.87]	1.03 [0.49–1.63]

**Table A4:** Per-class confusion counts of the detector against *each* clinician over all 621 repetitions (TP: both flag; FP: system only; FN: clinician only; TN: neither). These are the raw counts underlying the per-rater metrics of Table 8.

Compensation	vs. Rater 1				vs. Rater 2			
	TP	FP	FN	TN	TP	FP	FN	TN
Incomplete elbow extension	58	18	28	517	58	18	26	519
Inter-limb asymmetry	96	58	42	425	92	62	62	405
Shoulder elevation	83	65	69	404	67	81	53	420
Trunk lean	88	14	1	518	87	15	1	518
Head tilt	91	9	12	509	90	10	17	504

**Table A5:** Class prevalence (fraction of positive labels, averaged over the two raters) in the full cohort versus the consensus subset. The consensus subset, which drops the 144 disputed repetitions, has a lower positive share for every sign, most for trunk lean—confirming that it is an easier evaluation set than whole-cohort operation.

Compensation	Full (621)	Consensus (477)	Δ
Incomplete elbow extension	0.137	0.130	−0.007
Inter-limb asymmetry	0.235	0.197	−0.038
Shoulder elevation	0.219	0.214	−0.005
Trunk lean	0.143	0.090	−0.053
Head tilt	0.169	0.136	−0.033
